# Unpredictable cycloisomerization of 1,11-dien-6-ynes by a common cobalt catalyst

**DOI:** 10.3762/bjoc.13.62

**Published:** 2017-03-31

**Authors:** Abdusalom A Suleymanov, Dmitry V Vasilyev, Valentin V Novikov, Yulia V Nelyubina, Dmitry S Perekalin

**Affiliations:** 1 Nesmeyanov Institute of Organoelement Compounds, Russian Academy of Sciences, 28 Vavilova str., 119991, Moscow, Russian Federation

**Keywords:** catalysis, cobalt, cyclization, enynes, ligands

## Abstract

1,11-Dien-6-ynes undergo cycloisomerization in the presence of the cobalt catalytic system CoBr_2_/phosphine ligand/Zn/ZnI_2_ giving cyclohexene, diene or cyclopropane structures depending on the type of the phosphine ligand. This unpredictable behaviour suggests that, although the availability of the cobalt catalytic system is appealing, the development of well-defined catalysts is desirable for further progress.

## Introduction

**Scheme 1:**
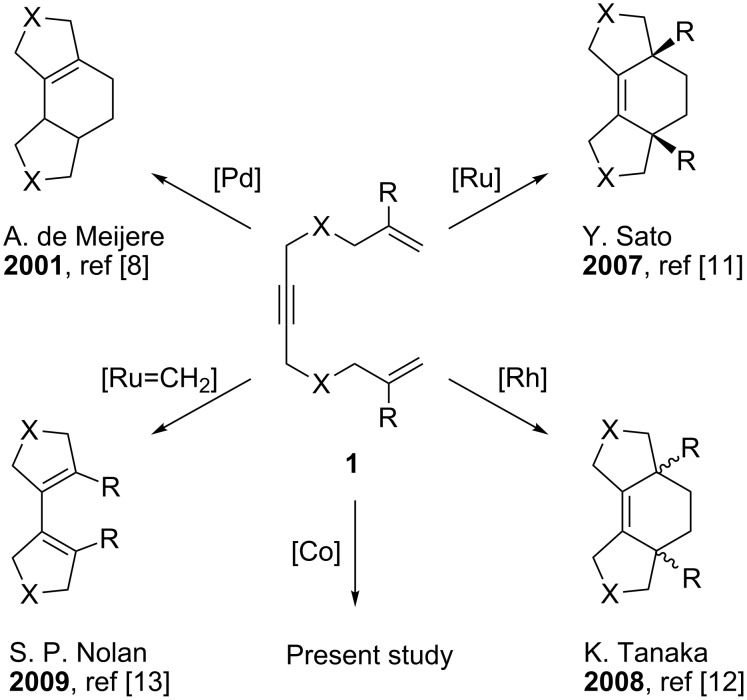
Examples of metal-catalyzed transformations of 1,11-dien-6-ynes.

Metal-catalyzed reactions of enynes represent an atom- and step-economical route to complex organic molecules with a broad range of functionalities [1–4]. In particular, cycloisomerization of enynes allows one to prepare compounds with exocyclic double bonds and cyclopropanes in a highly selective manner [5–7]. While catalytic transformations of enynes have been investigated in detail, there are only a few examples of similar reactions of dienynes [8–14]. Among dienynes, 1,11-dien-6-ynes **1** are of particular interest, because they are readily available in 1–2 steps from the commercial precursors (e.g., allyl bromides and 1,4-dibromo-2-butyne). Transformations of 1,11-dien-6-ynes provide an access to highly substituted polycycles [8,11–13] (Scheme 1). However, all these transformations require expensive noble metal catalysts with rather sophisticated ligands. Therefore, the development of a cheap catalyst for such reactions is highly desirable.

Over the last decade the application of the cobalt catalytic system CoBr_2_/phosphine ligand/Zn/ZnI_2_ for the carbon–carbon bond formation has become a subject of a growing interest (for reviews see [15–21]). This catalytic system has many advantages, including the availability of the cobalt salts and high tolerance to organic functional groups (for some recent examples see [22–27]). However, it has been noted that the direction of the catalytic reactions often depends on the structure of the phosphine ligand. Major research on these ligand-controlled reactions has been made by the groups of Hilt and Cheng, in particular, on the selective formation of *meta*/*para*-products of Diels–Alder reactions [28–32], linear/branched products of hydrovinylation reactions [33–34], [2 + 2] cycloadditions vs Alder-ene reactions [35], *E*/*Z* isomerizations of alkenes [36–37] and other processes [38–42].

Herein, we report the cycloisomerizations of 1,11-dien-6-ynes in the presence of a cobalt catalytic system. We have found that the regioselectivity of these reactions dramatically and unpredictably depends on the structure of the phosphine ligand and on the structure of the substrate.

## Results and Discussion

**Scheme 2:**
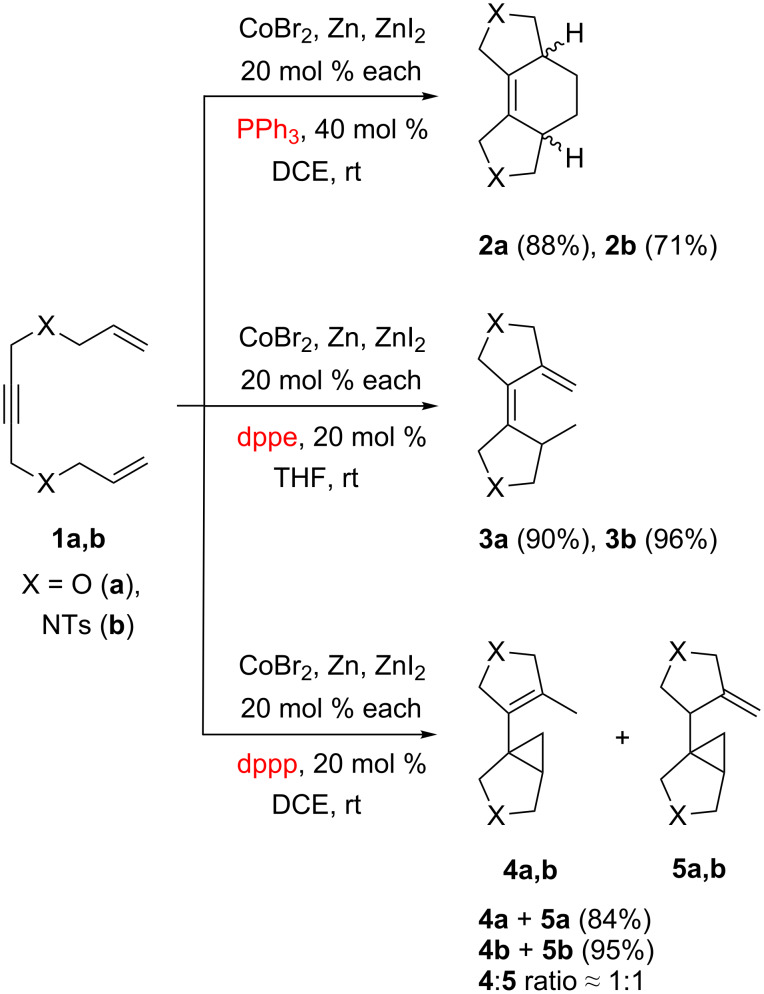
Cobalt-catalyzed cycloisomerizations of 1,11-dien-6-ynes.

First, we chose 1,4-bis(allyloxy)but-2-yne (**1a**) and 1,4-di(*N*-allyltosylamido)-2-butyne (**1b**) as test substrates for our investigation (Scheme 2). It was found that in the presence of CoBr_2_, Zn and ZnI_2_ (20 mol % each) as well as the triphenylphosphine ligand (40 mol %) in 1,2-dichloroethane (DCE) these substrates underwent clean [2 + 2 + 2]-cyclotrimerization to give cyclohexene derivatives **2a**,**b** in 70–90% yields. The rate of the reaction notably varied from experiment to experiment, but full conversion of **1a**,**b** into **2a**,**b** was usually achieved after 24 h. Attempts to employ 10 mol % loading of the pre-catalysts often led to incomplete conversion. Apparently, because of the heterogeneous nature of the mixture, only a small fraction of the cobalt salt actually formed the catalytically active species. Moreover, we found that it was important to activate the Zn/ZnI_2_ mixture prior addition of the substrate either by heating or by generation of ZnI_2_ in situ from the excess of Zn and I_2_. Variation of the solvent (MeCN, THF, DCE) and the reaction temperature (20–80 °C) had no significant influence on the reaction outcome.

Although the yields of **2a**,**b** were good, the compounds were formed as roughly 1:1 mixtures of *cis*- and *trans*-isomers (*cis*-isomers were reported previously [11] which helped us to assign the structures of **2a**,**b**). In attempt to improve the selectivity we used 1,2-bis(diphenylphosphino)ethane (dppe) as ligand instead of PPh_3_. However, this ligand completely changed the direction of the cyclization, converting **1a**,**b** into the diene compounds **3a,b** in 90–95% yields. These compounds were also obtained using a 1,2-bis(diphenylphosphino)benzene (dppbz) ligand. Despite the extensive studies of enyne transformations, this type of cyclization has been achieved only recently, using a cyclobutadiene rhodium complex as a catalyst [43].

Next, we employed 1,3-bis(diphenylphosphino)propane (dppp) as a phosphine ligand, which surprisingly led to conversion of **1** into some new isomeric products. The ^1^H NMR spectra of these products contained a number of signals in the 0.5–0.8 ppm region, which is characteristic for cyclopropane protons. On the basis of ^1^H–^1^H and ^1^H–^13^C correlation NMR spectra as well as GC–MS analysis we had assigned the structures of these products as the cyclopropane derivatives with internal **4** and exocyclic **5** double bonds (Scheme 2). Our spectral data correlate well with that of similar compounds, which were recently prepared by the rhodium-catalyzed cyclopropanation [44]. Compounds **4** and **5** were obtained as mixtures with approximately 1:1 ratio and total 80–90% yields. Numerous attempts to convert **5** into the more stable isomer **4** using strong bases or transition metal catalysts were unsuccessful. The cobalt catalytic system with other phosphine ligands, such as dppm, dppf, or BINAP, did not catalyze cycloisomerization of **1** into any products.

**Scheme 3:**
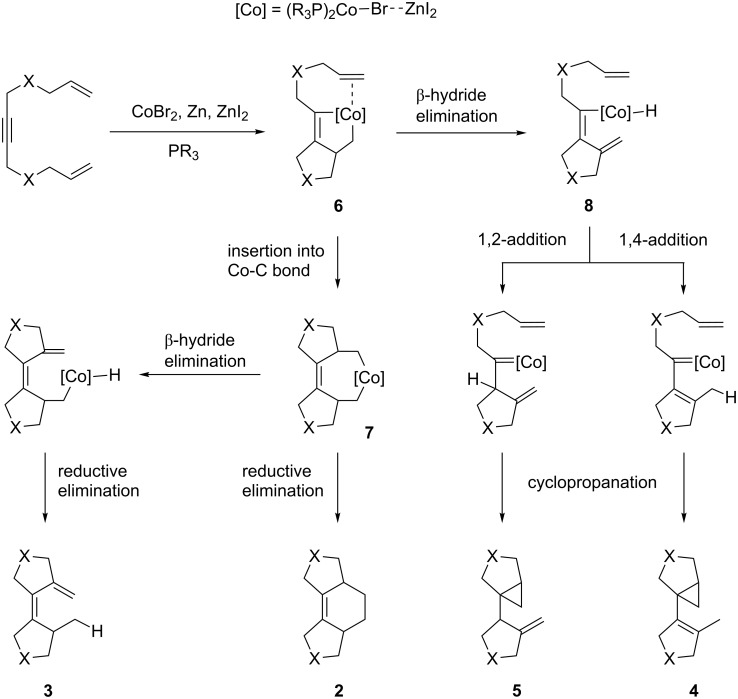
Possible mechanism for formation of the compounds **2**–**5**.

In order to explain the formation of products **2**–**5** we proposed a possible mechanism (Scheme 3) in accordance with the generally accepted concepts [15,18]. The crucial step of this mechanism is the formation of metallacycle **6** from unsaturated cobalt(I) species [Co(PR_3_)_2_Br] and the double and the triple bond of dienyne **1** (see Supporting Information File 1 for the calculated structure of **6**). Subsequent insertion of the double bond into the Co–C bond of **6** leads to the 7-membered metallacycle **7**, which can undergo reductive elimination to give the cyclohexene **2**, or β-hydride elimination followed by the reductive elimination to give the diene **3**. It may be speculated that the steric crowding in the intermediate **7** determines the direction of the reaction. In contrast to the relatively small dppe ligand, two large Ph_3_P ligands hinder the β-elimination (which requires proximal syn-position of metal and hydrogen) and therefore push reaction towards direct reductive elimination to give **2**.

Alternatively, β-hydride elimination can proceed in the intermediate **6** to give the hydride species **8**. Further, intramolecular 1,2- or 1,4-hydrometallation in **8** produces carbene complexes, which give cyclopropane derivatives **4** and **5**.

**Scheme 4:**
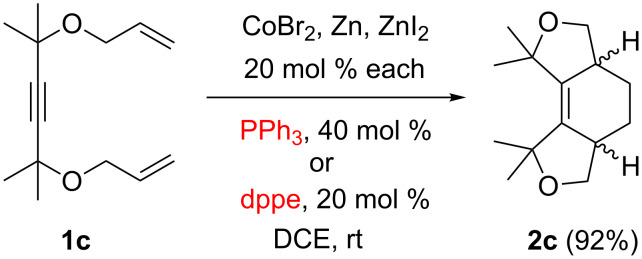
Cycloisomerization of the substituted dienyne **1c**.

In order to broaden the substrate scope, we studied similar reactions of the tetramethyl-substituted dienyne **1c** (Scheme 4). As expected, it produced the cyclohexene derivative **2c** in the presence of the triphenylphosphine ligand. More surprisingly, the same product was obtained when dppe or dppp were used as ligands, although the small amounts of isomeric by-products were detected in these cases. Noteworthy, according to the NMR spectra, product **2c** was formed as a single diastereomer, however, it was not possible to unambiguously determine whether it was the *cis*- or the *trans*-isomer by NMR (including various correlation techniques) because of the high symmetry of both possible isomers. Attempts to grow crystals of **2c** or its Br_2_ adduct for X-ray crystallography were also unsuccessful.

**Figure 1:**
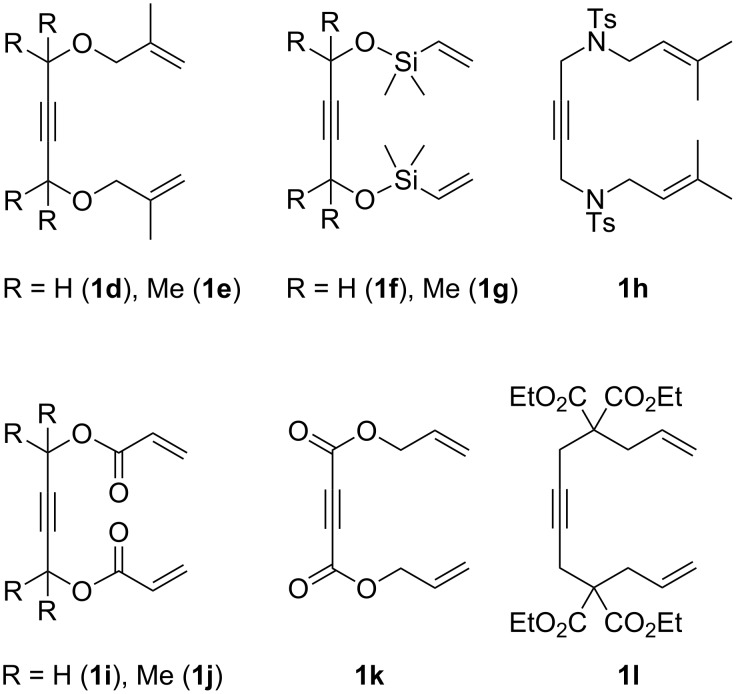
The substituted 1,11-dien-6-ynes that did not undergo cycloisomerization in the presence of the cobalt catalytic system.

Further experiments have shown that many other 1,11-dien-6-ynes **1d**–**l** (Figure 1) did not undergo cycloisomerization in the presence of the cobalt catalytic system regardless of the employed ligand. In the case of the substrates **1d**–**h** this may be explained by the increased steric crowding, which is evident from the 3D structure of the proposed intermediate **6** (see Supporting Information File 1). In the case of **1i**–**k** the electron acceptor effects or the restricted rotation of conjugated enones could be the problem. The case of dienyne **1l** was particularly surprising as it seemed to have a very similar structure to that of **1a**,**b**. We can speculate that COOEt groups of **1l** formed a chelate complex with ZnI_2_ and therefore strongly diminished its Lewis acidity, which is necessary for catalyst activation.

All the compounds obtained were characterized by ^1^H and ^13^C NMR spectroscopy as well as high-resolution mass-spectrometry (for ethers, which are oils) or elemental analysis (for tosylamides, which are crystalline solids). The structures of the starting dienyne **1h** and the cyclohexene *trans*-**2b** were established by X-ray crystallography. The structures of *cis*-**2b** and **3b** had been reported previously [11,43].

## Conclusion

To conclude, we have shown that the behavior of the popular catalytic system CoBr_2_/phosphine ligand/Zn/ZnI_2_ is very sensitive to the structure of the phosphine ligand and to the structure of the substrate. On the one hand, this allows one to discover unusual transformations of the unsaturated substrates. On the other hand, such sensitivity hinders the development of general methods, which can be applied to a wide range of compounds. Overall, although the availability of the cobalt catalytic system is appealing, the development of a well-defined catalyst with precisely-controlled ligand environment is necessary for further progress.

## Supporting Information

Experimental details and detailed spectroscopic data of all compounds are available as Supporting Information. Single crystal data for **1h** and **2b** (CCDC 1516680 and 1516681) has been deposited in the Cambridge Crystallographic Data Center.

File 1 Experimental details and detailed spectroscopic data of all compounds.

File 2 Single crystal data for **1h** and **2b**.

## References

[1] Michelet V, Toullec P Y, Genêt J-P (2008). Angew Chem, Int Ed.

[2] Jiménez-Núñez E, Echavarren A M (2008). Chem Rev.

[3] Domínguez G, Pérez-Castells J (2011). Chem Soc Rev.

[4] Domínguez G, Pérez-Castells J (2016). Chem – Eur J.

[5] Bruneau C (2005). Angew Chem, Int Ed.

[6] Inglesby P A, Evans P A (2010). Chem Soc Rev.

[7] Amatore M, Aubert C (2015). Eur J Org Chem.

[8] van Boxtel L J, Körbe S, Noltemeyer M, de Meijere A (2001). Eur J Org Chem.

[9] Himes R A, Fanwick P E, Rothwell I P (2003). Chem Commun.

[10] Shibata T, Tahara Y-k (2006). J Am Chem Soc.

[11] Tanaka D, Sato Y, Mori M (2007). J Am Chem Soc.

[12] Sagae H, Noguchi K, Hirano M, Tanaka K (2008). Chem Commun.

[13] Clavier H, Correa A, Escudero-Adán E C, Benet-Buchholz J, Cavallo L, Nolan S P (2009). Chem – Eur J.

[14] Saito N, Tanaka D, Mori M, Sato Y (2011). Chem Rec.

[15] Jeganmohan M, Cheng C-H (2008). Chem – Eur J.

[16] Hess W, Treutwein J, Hilt G (2008). Synthesis.

[17] Cahiez G, Moyeux A (2010). Chem Rev.

[18] Gao K, Yoshikai N (2014). Acc Chem Res.

[19] Pellissier H, Clavier H (2014). Chem Rev.

[20] Gandeepan P, Cheng C-H (2015). Acc Chem Res.

[21] Röse P, Hilt G (2016). Synthesis.

[22] Saino N, Amemiya F, Tanabe E, Kase K, Okamoto S (2006). Org Lett.

[23] Sakurada T, Sugiyama Y-k, Okamoto S (2013). J Org Chem.

[24] Geny A, Gaudrel S, Slowinski F, Amatore M, Chouraqui G, Malacria M, Aubert C, Gandon V (2009). Adv Synth Catal.

[25] Achard M, Tenaglia A, Buono G (2005). Org Lett.

[26] Toselli N, Martin D, Achard M, Tenaglia A, Bürgi T, Buono G (2008). Adv Synth Catal.

[27] Nishimura A, Tamai E, Ohashi M, Ogoshi S (2014). Chem – Eur J.

[28] Hilt G, Korn T J (2001). Tetrahedron Lett.

[29] Hilt G, Janikowski J, Hess W (2006). Angew Chem, Int Ed.

[30] Kuttner J R, Warratz S, Hilt G (2012). Synthesis.

[31] Fiebig L, Kuttner J, Hilt G, Schwarzer M C, Frenking G, Schmalz H-G, Schäfer M (2013). J Org Chem.

[32] Hilt G, Janikowski J, Schwarzer M, Burghaus O, Sakow D, Bröring M, Drüschler M, Huber B, Roling B, Harms K (2014). J Organomet Chem.

[33] Arndt M, Dindaroğlu M, Schmalz H-G, Hilt G (2011). Org Lett.

[34] Mannathan S, Cheng C-H (2012). Chem – Eur J.

[35] Hilt G, Paul A, Treutwein J (2010). Org Lett.

[36] Pünner F, Schmidt A, Hilt G (2012). Angew Chem, Int Ed.

[37] Schmidt A, Nödling A R, Hilt G (2015). Angew Chem, Int Ed.

[38] Chang H-T, Thiruvellore T J, Wang C-C, Cheng C-H (2007). J Am Chem Soc.

[39] Wei C-H, Mannathan S, Cheng C-H (2011). J Am Chem Soc.

[40] Wei C-H, Mannathan S, Cheng C-H (2012). Angew Chem, Int Ed.

[41] Santhoshkumar R, Mannathan S, Cheng C-H (2014). Org Lett.

[42] Santhoshkumar R, Mannathan S, Cheng C-H (2015). J Am Chem Soc.

[43] Perekalin D S, Shvydkiy N V, Nelyubina Y V, Kudinov A R (2015). Chem – Eur J.

[44] Torres O, Roglans A, Pla-Quintana A (2016). Adv Synth Catal.

