# Supplementary material for: Unpredictable cycloisomerization of 1,11-dien-6-ynes by a common cobalt catalyst
Source: Beilstein J Org Chem. 2017 Mar 31;13:639–43. doi: 10.3762/bjoc.13.62 (PMC5389203; doi:10.3762/bjoc.13.62)
Supplement: File 1 — Experimental details and detailed spectroscopic data of all compounds. [file Beilstein_J_Org_Chem-13-639-s001.pdf]

Supporting Information

for

Unpredictable cycloisomerization of 1,11-diene-6-ynes by a common cobalt catalyst

Abdusalom A. Suleymanov[1], Dmitry V. Vasilyev[1], Valentin V. Novikov[1], Yulia V. Nelyubina[1], Dmitry S. Perekalin[*1]

Address: [1]Nesmeyanov Institute of Organoelement Compounds, Russian Academy of Sciences, 28 Vavilova str., 119991, Moscow, Russian Federation

Email: Dmitry S. Perekalin - dsp@ineos.ac.ru

*Corresponding author

Experimental details and detailed spectroscopic data of all compounds

Content:

1. General information S2

2. General procedure for the cobalt-catalyzed cycloisomerization reactions S2

3. NMR spectra and related data S8

4. X-ray experimental details and structures S18

5. Optimized structure of the suggested key intermediate **6** S20

General information

All reactions were carried out under an argon atmosphere using Schlenk technique. The solvents were purified and dried using standard methods. All reagents have commercial origin. In particular, Zn dust (<10 µm, ≥98%) was obtained from Aldrich (product # 209988). Starting 1,11-diene-6-ynes **1a**–**1l** were synthesized similar to the literature procedures (H. Clavier, A. Correa, E.C. Escudero-Adan, B.-B. Jordi, L. Cavallo, S.P. Nolan, *Chem. Eur. J.*, **2009**, *15*, 10244). Column chromatography was performed using Macherey-Nagel silica gel (size: 230−400 mesh). Analysis of TLC plates was carried out using a concentrated $KMnO_4$ solution as a stain or by UV lamp (254 nm). NMR spectra were measured using Bruker Avance 400 and Bruker Avance 600 spectrometers. Peaks were aligned with respect to residual signal of deuterated chloroform (^{1}H: δ 7.26 ppm; ^{13}C: δ 77.16 ppm). HRMS data were acquired using electrospray (ESI) ionization and time-of-flight (TOF) detection.

General procedure for the cobalt-catalyzed cycloisomerization reactions

Important note: Due to the high sensitivity of the catalyst, the loading of reagents should be carried out as written below. Attempts to run the reaction by simple mixing of all reactants without prior activation of Zn / ZnI_2 system usually gave no product.

Activation methods of Zn + ZnI_2:

Method a: Zn dust (0.4 equiv) and iodine (0.2 equiv) were mixed in an appropriate solvent and the mixture was stirred vigorously at room temperature until disappearance of iodine color (usually overnight). Ultrasonic activation of the mixture sometimes helped to accelerate the reaction.

Method b: Zn dust (0.2 equiv) and ZnI_2 (0.2 equiv) were mixed and heated as solid at about 200 °C by heatgun under high vacuum for 10–20 min and then allowed to cool to room temperature.

Method c: Suspension of Zn dust (0.2 equiv) and ZnI_2 (0.2 equiv) in a solvent was refluxed for 3-5 minutes under vigorous stirring and then allowed to cool to room temperature.

Example of procedure for cycloisomerization of **1b** into **3b**: In a Schlenk-tube under argon atmosphere a suspension of Zn dust (0.1 mmol, 6.5 mg) and ZnI_2 (0.1 mmol, 32 mg) in 4 ml of THF was refluxed for 5 minutes with stirring for activation and then allowed to cool to room temperature. Then dppe (0.1 mmol, 40 mg), $CoBr_2$ (0.1 mmol, 22 mg) and **1b** (0.5 mmol, 236 mg) were added. The mixture was stirred (600 rpm) overnight. The solvent was removed in vacuum and the residue was purified by flash chromatography using mixture of petroleum ether (40-60) and ethyl acetate (ratio 5:1) as eluent.

1,3,3a,4,5,5a,6,8-Octahydrobenzo[1,2-*c*:3,4-*c'*]difuran (**2a**)

(*cis*-isomer was previously reported: D. Tanaka, Y. Sato M. Mori *J. Am. Chem. Soc.*, **2007**, *129*, 7730)

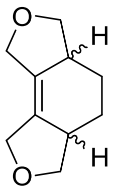

2a

Colorless oil. Yield 78 mg (88%).

The product was synthesized following the general procedure from 2,5-bis(allyloxy)but-2-yne (0.5 mmol, 88 mg) using PPh_3 (0.2 mmol, 52 mg) as a ligand and 1,2-dichloroethane (4 mL) as solvent.

$\mathbf{R_f}$ (PE/EA=5/1): 0.31; **^{1}H NMR** (400 MHz, $CDCl_3$): δ 4.34-4.16 (m, 6H, 2 x CH_2O + 2 x OCH), 3.22-3.17 (m, 2H, 2 x CHO), 2.63-2.56 (br m, 2H, 2 x CH), 2.10-2.07 (m, 1H, $\underline{CH_2}CH_{2(trans)}$), 1.85-1.78 (m, 1H, $\underline{CH_2}CH_{2(cis)}$), 1.27-1.21 (m, 2H, CH_2CH_2). **^{13}C NMR** (101 MHz, $CDCl_3$): δ 131.6 (2 x $C_{sp2(cis)}$), 130.2 (2 x $C_{sp2(trans)}$), 73.7 (2 x $CH_2O_{(trans)}$), 73.06 (2 x $CH_2O_{(cis)}$), 68.1 (2 x $CH_2O_{(cis)}$), 67.5 (2 x $CH_2O_{(trans)}$), 41.4 (2 x $CH_{(trans)}$), 38.09 (2 x $CH_{(cis)}$), 24.4 ($CH_2CH_{2(trans)}$), 22.7 ($CH_2CH_{2(cis)}$). ^{1}H and ^{13}C NMR signals of *cis*-isomer match with the literature data. **HRMS** (ESI): Simulated $C_{10}H_{15}O_2^+$ (MH^+) 167.1072; Found 167.1067.

(Z)-4-Methyl-4'-methylene-4,4',5,5'-tetrahydro-2*H*,2'*H*-3,3'-bifuranylidene (**3a**)

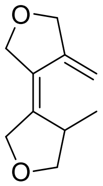

3a

Colorless oil. Yield 79 mg (90%).

The product was synthesized following the general procedure from 2,5-bis(allyloxy)but-2-yne (0.5 mmol, 88 mg) using dppe (0.1 mmol, 41 mg) as ligand and THF (4 mL) as solvent.

$\mathbf{R_f}$ (PE/EA=4/1): 0.36; **^{1}H NMR** (400 MHz, $CDCl_3$): δ 5.18 (s, 1H, $CH_{(methylene)}$), 5.04 (s, 1H, $CH_{(methylene)}$), 4.44–4.41 (m, 2H, CH_2), 4.30–4.25 (m, 3H, NCH_2 + NCH), 4.15–4.08 (m, 1H, NCH), 3.80–3.75 (m, 2H, CH_2), 3.10 (m, 1H, CH), 1.15 (d, J = 7.0 Hz, 3H, CH_3); **^{13}C NMR** (101 MHz, $CDCl_3$): δ 143.7 (C_{sp2}), 138.7 (C_{sp2}), 125.3 (C_{sp2}), 105.1 ($CH_{2(methylene)}$), 76.0 (CH_2), 74.0 (CH_2), 72.0 (CH_2), 70.4 (CH_2), 36.5 (CH), 16.3 (CH_3). **HRMS** (ESI): Simulated $C_{10}H_{15}O_2^+$ (MH^+) 167.1072; Found 167.0977.

1-(4-Methyl-2,5-dihydrofuran-3-yl)-3-oxabicyclo[3.1.0]hexane (**4a**)
and 1-(4-methylenetetrahydrofuran-3-yl)-3-oxabicyclo[3.1.0]hexane (**5a**)

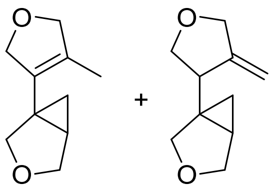

4a **5a**

Colorless oil with fruity smell. Yield 70 mg (84%).

The product was synthesized following the general procedure from 2,5-bis(allyloxy)but-2-yne (0.5 mmol, 88 mg) using dppp (0.1 mmol, 41 mg) as ligand and 1,2-dichloroethane (4 mL) as solvent.

R_f (PE/EA=5/2): 0.37; **^{1}H NMR** (400 MHz, $CDCl_3$): δ 5.04–5.01 (m, 2H, $CH_{2(methylene)}$), 4.59–4.53 (m, 2H, OCH_2), 4.53–4.44 (m, 6H, 3 x OCH_2), 4.27–4.25 (m, 2H, OCH_2), 3.95–3.93 (m, 1H, OCH), 3.86–3.84 (m, 2H, OCH_2), 3.82–3.80 (m, 2H, OCH_2), 3.76–3.71 (m, 5H, 2 x OCH_2 + OCH), 3.64–3.57 (m, 4H, 2 x OCH_2), 2.69–2.64 (m, 1H, CH), 1.69–1.67 (s, 6H, CH_3), 1.56–1.47 (m, 3H, 3 x $CH_{cyclopropane}$), 0.78–0.76 (m, 2H, 2 x $CH_{cyclopropane}$), 0.73–0.70 (m, 2H, 2 x $CH_{cyclopropane}$), 0.60–0.57 (m, 1H, $CH_{cyclopropane}$), 0.55–0.52 (m, 1H, $CH_{cyclopropane}$); **^{13}C NMR** (101 MHz, $CDCl_3$): δ 149.8 ($C_{sp2(methylene)}$), 131.7 (C_{sp2}), 128.3 (C_{sp2}), 105.4 ($CH_{2(methylene)}$), 80.3 (OCH_2), 77.6 (OCH_2), 72.7 (OCH_2), 72.0 (OCH_2), 71.5 (OCH_2), 70.3 (OCH_2), 69.8 (OCH_2), 69.7 (OCH_2), 46.0 (CH), 30.0 ($C_{cyclopropane}$), 24.6 ($C_{cyclopropane}$), 22.7 ($CH_{cyclopropane}$), 22.3 ($CH_{2cyclopropane}$), 12.7 ($CH_{2(cyclopropane)}$), 11.4 ($CH_{2(cyclopropane)}$), 10.3 (CH_3). **HRMS** (ESI): Simulated $C_{10}H_{15}O_2^+$ (MH^+) 167.1072; Found 167.1067.

2,7-Ditosyl-1,2,3,3a,4,5,5a,6,7,8-decahydropyrrolo[3,4-*e*]isoindole (**2b**)
(*cis*-isomer was previously reported: D. Tanaka, Y. Sato M. Mori *J. Am. Chem. Soc.*, **2007**, *129*, 7730)

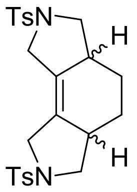

2b

Colorless solid. Yield 167 mg (71%).

The product was synthesized following the general procedure from *N*,*N'*-(but-2-yne-1,4-diyl)bis(*N*-allyl-4-methylbenzenesulfonamide) (0.5 mmol, 236 mg) using PPh_3 (0.15 mmol, 39 mg) as ligand and 1,2-dichloroethane (4 mL) as solvent. Crystals of trans-**2b** for X-Ray analysis were obtained by slow evaporation of solvent from PE/EA=1/1 solution.

R$_f$ (PE/EA=2/1): 0.39; **^{1}H NMR** (400 MHz, $CDCl_3$): δ 7.69 (d, *J* = 8.1 Hz, 4H, 4 x CH_{ar}), 7.31 (d, *J* = 8.0 Hz, 4H, 4 x CH_{ar}), 3.91 – 3.56 (m, 6H, 3 x NCH_2), 2.56 – 2.31 (m, 10H, 2 x CH_3 + 2 x CH + NCH_2), 1.76 – 1.56 (m, 2H, CH_2), 1.15 – 0.97 (m, 2H, CH_2); **^{13}C NMR** (101 MHz, $CDCl_3$): δ 143.9 (2 x $CH_3\underline{C_{ar}}$), 143.8 (2 x $CH_3\underline{C_{ar}}$), 133.7 (2 x $SO_2\underline{C_{ar}}$), 133.6 (2 x $SO_2\underline{C_{ar}}$), 130.4 (2 x $C_{sp2(cis)}$), 129.83 (2 x $CH_3C\underline{CH_{ar}}$), 129.81 (2 x $CH_3C\underline{CH_{ar}}$), 129.2 (2 x $C_{sp2(trans)}$), 127.7 (2 x $SO_2C\underline{CH_{ar}}$), 127.6 (2 x $SO_2C\underline{CH_{ar}}$), 53.9 (2 x $CH_2N_{(trans)}$), 52.8 (2 x $CH_2N_{(cis)}$), 49.2 (2 x $CH_2N_{(cis)}$), 48.7 (2 x $CH_2N_{(trans)}$), 39.9 (2 x $CH_{(trans)}$), 37.0 (2 x $CH_{(cis)}$), 25.0 ($CH_2CH_{2(trans)}$), 23.0 ($CH_2CH_{2(cis)}$), 21.7 (2 x CH_3). ^{13}C NMR signals of *cis*-isomer match with the literature data. **EA:** Found: % C 61.21, H 6.26, N 5.79. Calc. for $C_{24}H_{28}N_2O_4S_2$: % C 60.99, H 5.97, N 5.93.

(Z)-4-Methyl-4'-methylene-1,1'-ditosyl-1,1',2,2',4,4',5,5'-octahydro-3,3'-bipyrrolylidene (**3b**)
Previously reported: D.S. Perekalin, N.V. Shvydkiy, Y.V. Nelyubina, A.R. Kudinov, *Chem. Eur. J.* **2015**, *21*, 16344.

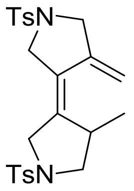

3b

Colorless solid. Yield 226 mg (96%).
The product was synthesized following the general procedure from *N*,*N*'-(but-2-yne-1,4-diyl)bis(*N*-allyl-4-methylbenzenesulfonamide) (0.5 mmol, 236 mg) using dppe (0.1 mmol, 40 mg) as ligand and THF (4 mL) as solvent.

R$_f$ (PE/EA=5/2): 0.21; **^{1}H NMR** (600 MHz, $CDCl_3$): δ 7.70 (d, J = 7.9 Hz, 2H), 7.67 (d, J = 7.9 Hz, 2H), 7.35 (d, J = 7.9 Hz, 2H), 7.28 (d, J = 7.9 Hz, 2H), 5.06 (s, 2H), 3.97 (d, *J* = 13.5 Hz, 1H), 3.91 (d, *J* = 15.4 Hz, 1H), 3.85 (d, *J* = 13.5 Hz, 1H), 3.75 (d, *J* = 13.5 Hz, 1H), 3.64 (d, *J* = 13.5 Hz, 1H), 3.38 (d, *J* = 15.4 Hz, 1H), 3.33 (d, *J* = 9.0 Hz, 1H), 3.03 – 2.96 (m, 1H), 2.96 – 2.92 (m, 1H), 2.43 (d, *J* = 15.6 Hz, 1H), 1.07 (d, *J* = 6.8 Hz, 3H); **^{13}C NMR** (151 MHz, $CDCl_3$): δ 140.00, 137.68, 129.75, 129.71, 127.84, 124.87, 109.18, 55.15, 54.16, 52.25, 51.26, 35.68, 21.48, 16.74; Detailed correlation of NMR signals with structure find below on page S14. **HRMS** (ESI): Simulated $C_{24}H_{29}N_2O_4S_2^+$ (MH^+) 473.1569, Found 473.1547; Simulated $C_{24}H_{28}N_2O_4S_2Na^+$ (MNa^+) 495.1388, Found 495.1359. **EA:** Found: % C 61.06, H 6.05, N 5.90. Calc. for $C_{24}H_{28}N_2O_4S_2$: % C 60.99, H 5.97, N 5.93.

1-(4-Methyl-1-tosyl-2,5-dihydro-1*H*-pyrrol-3-yl)-3-tosyl-3-azabicyclo[3.1.0]hexane (**4b**) and 1-(4-methylene-1-tosylpyrrolidin-3-yl)-3-tosyl-3-azabicyclo[3.1.0]hexane (**5b**)

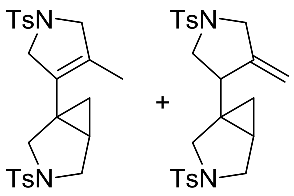

4b **5b**

Colorless solid. Yield 226 mg (95%).

The product was synthesized following the general procedure from *N*,*N'*-(but-2-yne-1,4-diyl)bis(*N*-allyl-4-methylbenzenesulfonamide) (0.5 mmol, 236 mg) using dppp (0.1 mmol, 41 mg) as ligand and 1,2-dichloroethane (4 mL) as solvent.

R_f (PE/EA=5/2): 0.13; **^{1}H NMR** (600 MHz, $CDCl_3$): δ 7.77 – 7.56 (m, 8H), 7.42 – 7.21 (m, 8H), 4.90 (d, *J* = 2.1 Hz, 1H), 4.72 (d, *J* = 2.3 Hz, 1H), 4.01 – 3.87 (m, 3H), 3.81 (s, 2H), 3.67 (d, *J* = 14.0 Hz, 1H), 3.54 (dd, *J* = 9.2, 6.0 Hz, 2H), 3.48 (d, *J* = 9.5 Hz, 1H), 3.38 (dd, *J* = 9.4, 7.9 Hz, 1H), 3.32 (d, *J* = 9.3 Hz, 1H), 3.14 (dd, *J* = 9.5, 3.9 Hz, 1H), 3.06 (dd, *J* = 9.3, 3.7 Hz, 1H), 2.99 (d, *J* = 9.3 Hz, 1H), 2.85 – 2.76 (m, 2H), 2.58 – 2.38 (m, 14H), 1.57 (s, 3H), 1.37 (tt, *J* = 12.7, 6.4 Hz, 2H), 0.91 – 0.80 (m, 2H), 0.65 (dd, *J* = 8.0, 5.0 Hz, 1H), 0.54 (dd, *J* = 7.8, 5.5 Hz, 1H), 0.46 (t, *J* = 4.8 Hz, 1H); **^{13}C NMR** (151 MHz, $CDCl_3$): δ 145.12, 144.36, 144.10, 143.91, 143.87, 134.28, 133.98, 133.45, 132.61, 132.51, 130.15, 130.10, 130.07, 129.95, 128.22, 128.10, 127.84, 127.74, 127.71, 127.66, 108.93, 60.71, 59.31, 56.44, 52.72, 52.09, 51.78, 50.40, 50.19, 49.87, 46.39, 28.41, 24.20, 21.91, 21.89, 21.86, 21.74, 21.67, 14.50, 14.11, 12.46, 12.25; Detailed correlation of NMR signals with structure find below on pages S16-17. **HRMS** (ESI): Simulated $C_{24}H_{29}N_2O_4S_2^+$ (MH^+) 473.1569; Found 473.1515. **EA:** Found: % C 60.82, H 5.81, N 5.76. Calc. for $C_{24}H_{28}N_2O_4S_2$: % C 60.99, H 5.97, N 5.93.

1,1,8,8-Tetramethyl-1,3,3a,4,5,5a,6,8-octahydrobenzo[1,2-*c*:3,4-*c*']difuran (**2c**)

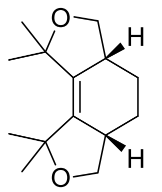

2c

Colorless oil. Yield 102 mg (92%).

The product was synthesized following the general procedure from 2,5-bis(allyloxy)-2,5-dimethylhex-3-yne (0.5 mmol, 111 mg) using dppp (0.1 mmol, 41 mg) as ligand and 1,2-dichloroethane (4 mL) as solvent.

R_f (PE/EA=5/1): 0.31; **^{1}H NMR** (400 MHz, $CDCl_3$): δ 4.11 (dd, *J* = 8.4, 8.4 Hz, 2H, 2 x OCH), 3.44 (dd, *J* = 8.7, 8.7 Hz, 2H, 2 x OCH), 2.59–2.42 (m, 2H, 2 x CH), 1.91–1.76 (m, 2H, CH_2), 1.43

(s, 6H, 2 x CH_3), 1.41 (s, 6H, 2 x CH_3), 1.22–1.07 (m, 2H, CH_2); **^{13}C NMR** (101 MHz, $CDCl_3$): δ 138.8 (2 x C_{sp2}), 81.1 (2 x CMe_2), 70.2 (2 x OCH_2), 42.3 (2 x CH), 28.2 (2 x CH_3), 27.5 (2 x CH_3), 24.5 (2 x CH_2). **HRMS** (ESI): Simulated $C_{14}H_{23}O_2^+$ (MH^+) 223.1698 ; Found 223.1693.

NMR spectra

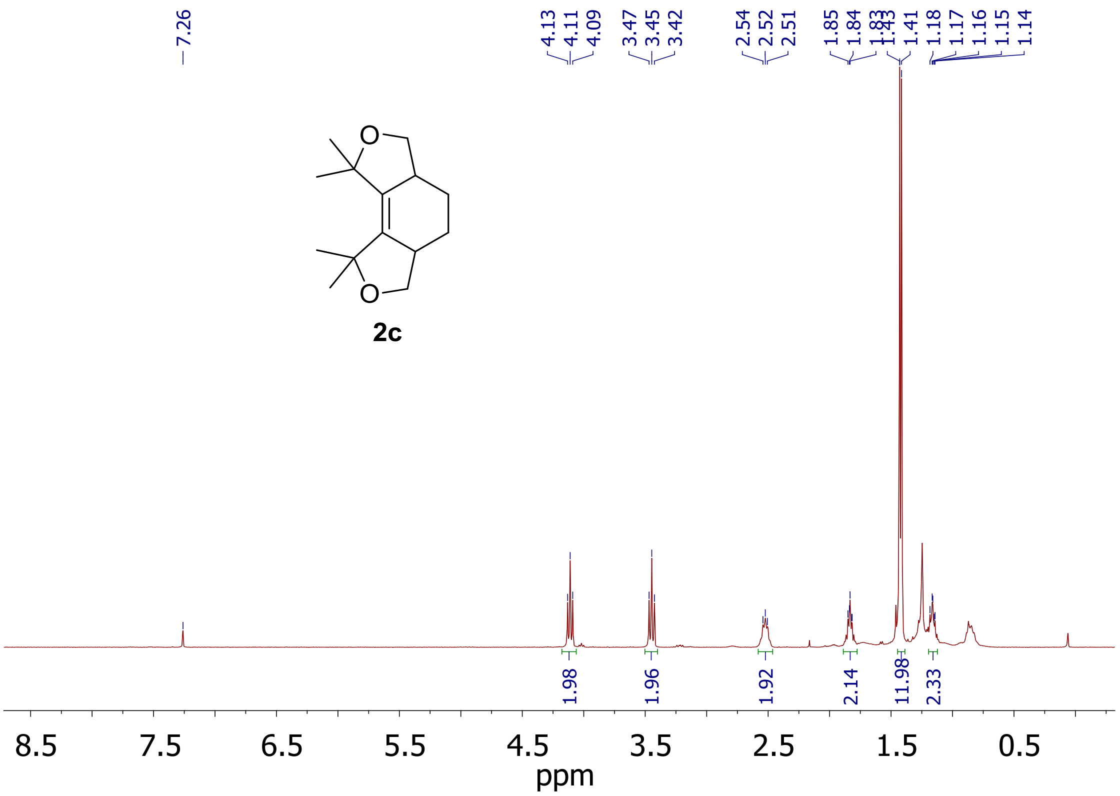

Figure S1. ^{1}H NMR spectrum of **2c** (400 MHz, $CDCl_3$).

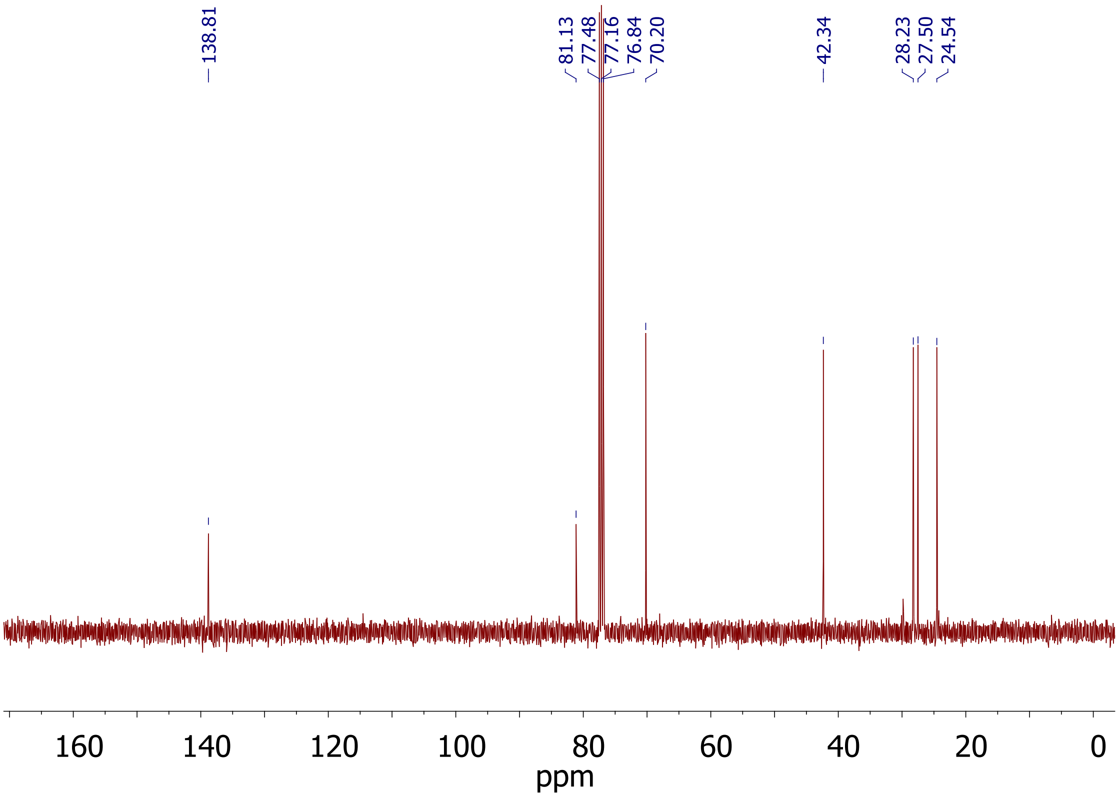

Figure S2. ^{13}C NMR spectrum of **2c** (101 MHz, $CDCl_3$).

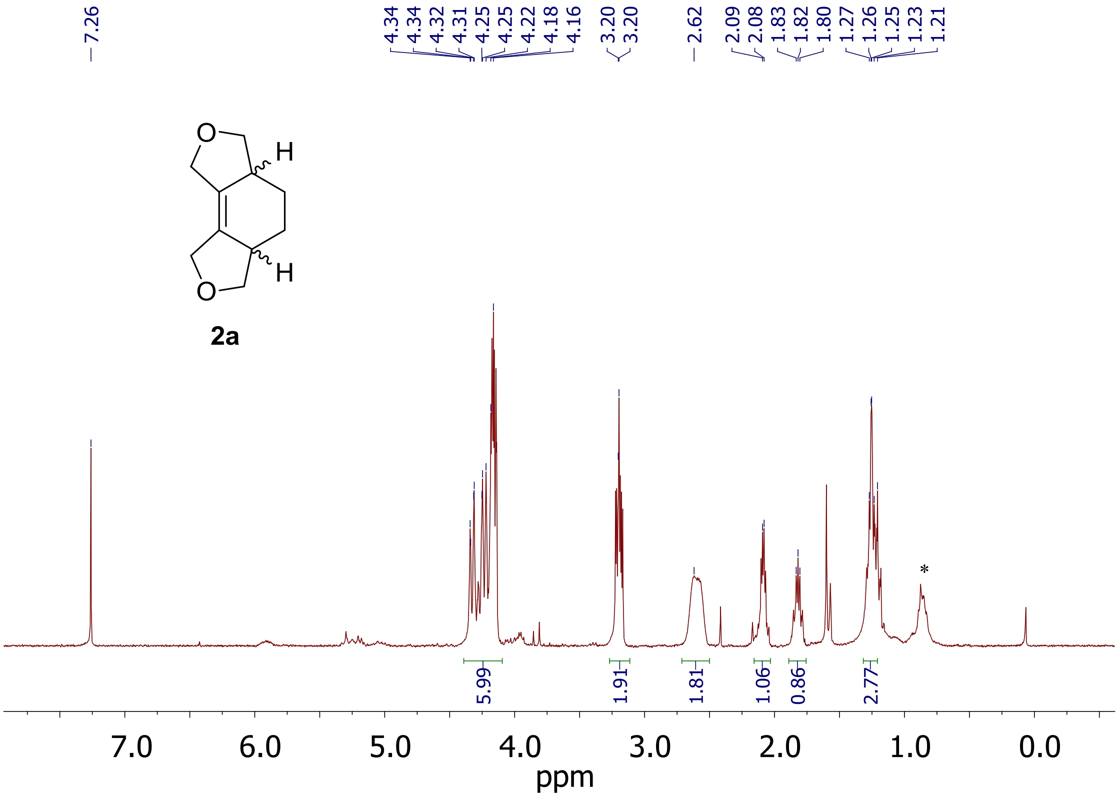

Figure S3. ^{1}H NMR spectrum of **2a** (400 MHz, $CDCl_3$). * - grease

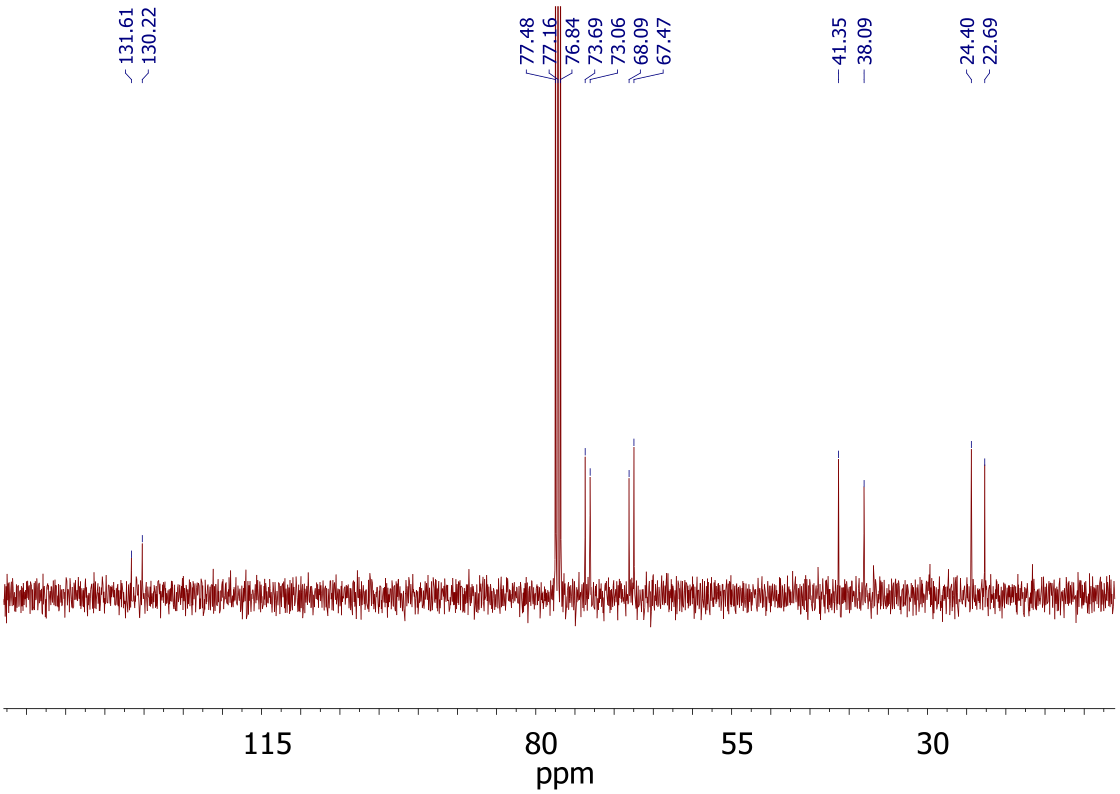

Figure S4. ^{13}C NMR spectrum of **2a** (101 MHz, $CDCl_3$).

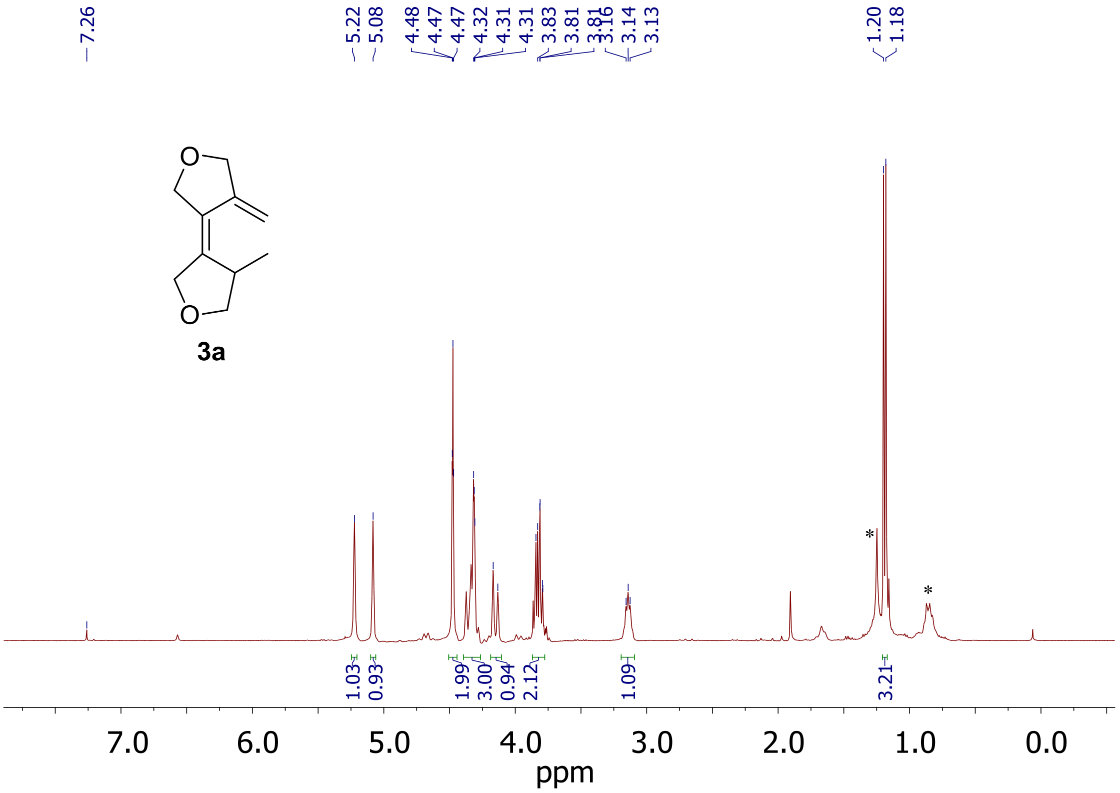

Figure S5. ^{1}H NMR spectrum of **3a** (400 MHz, $CDCl_3$). * - grease

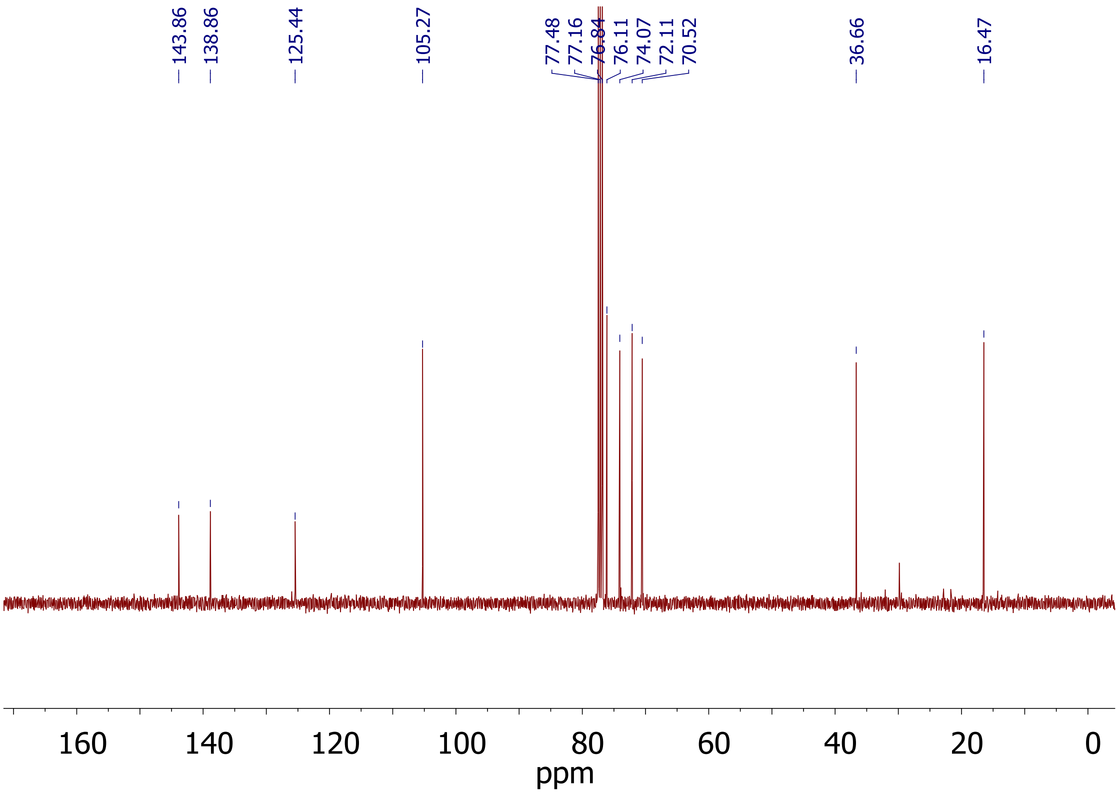

Figure S6. ^{13}C NMR spectrum of **3a** (101 MHz, $CDCl_3$).

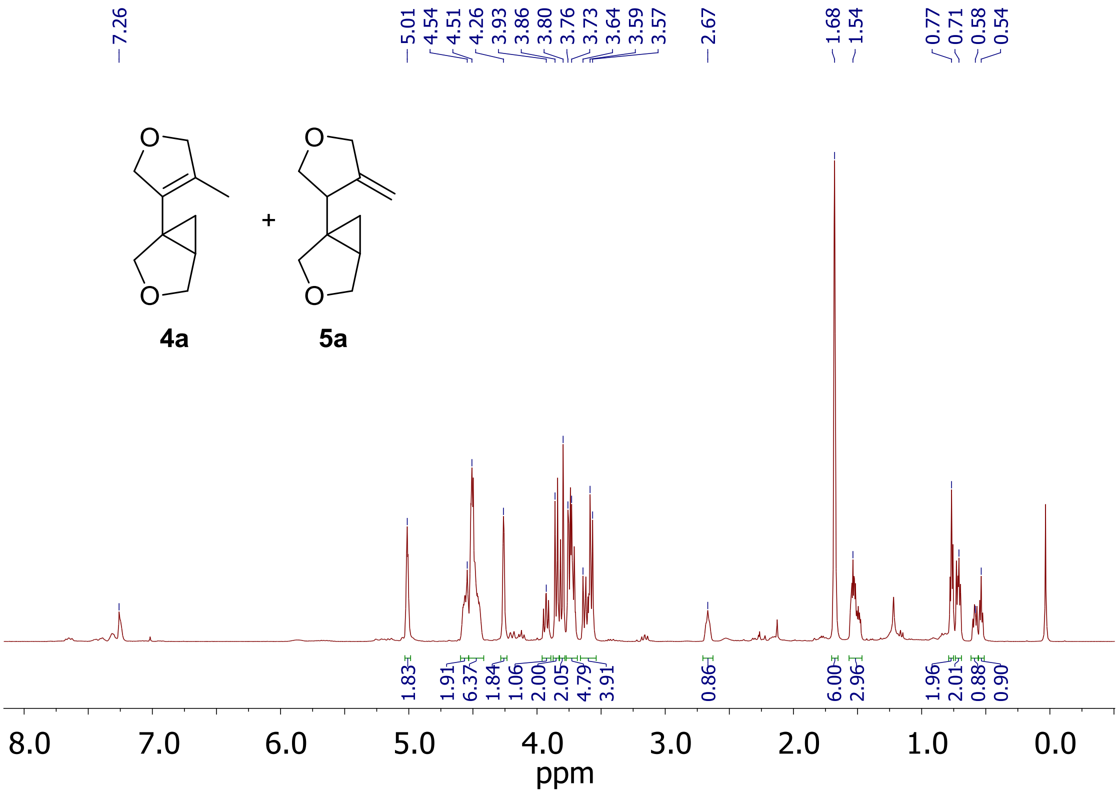

Figure S7. ^{1}H NMR spectrum of mixture **4a** and **5a** (400 MHz, $CDCl_3$).

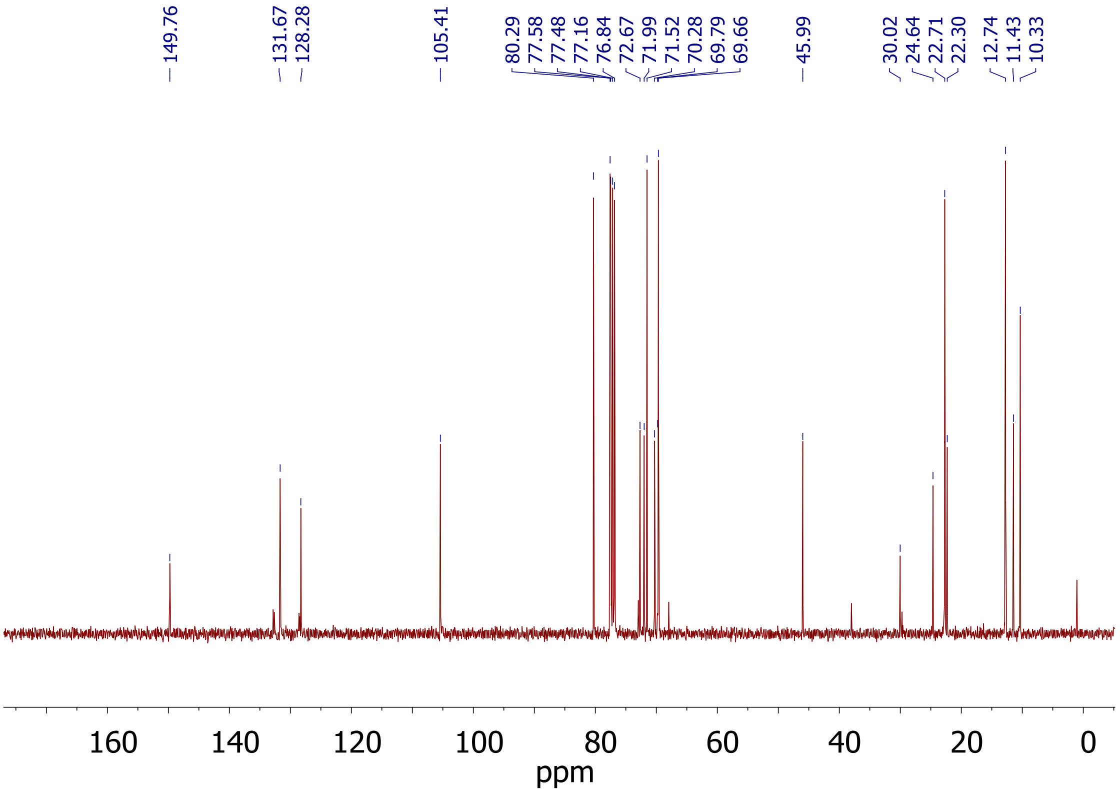

Figure S8. ^{13}C NMR spectrum of mixture **4a** and **5a** (101 MHz, $CDCl_3$).

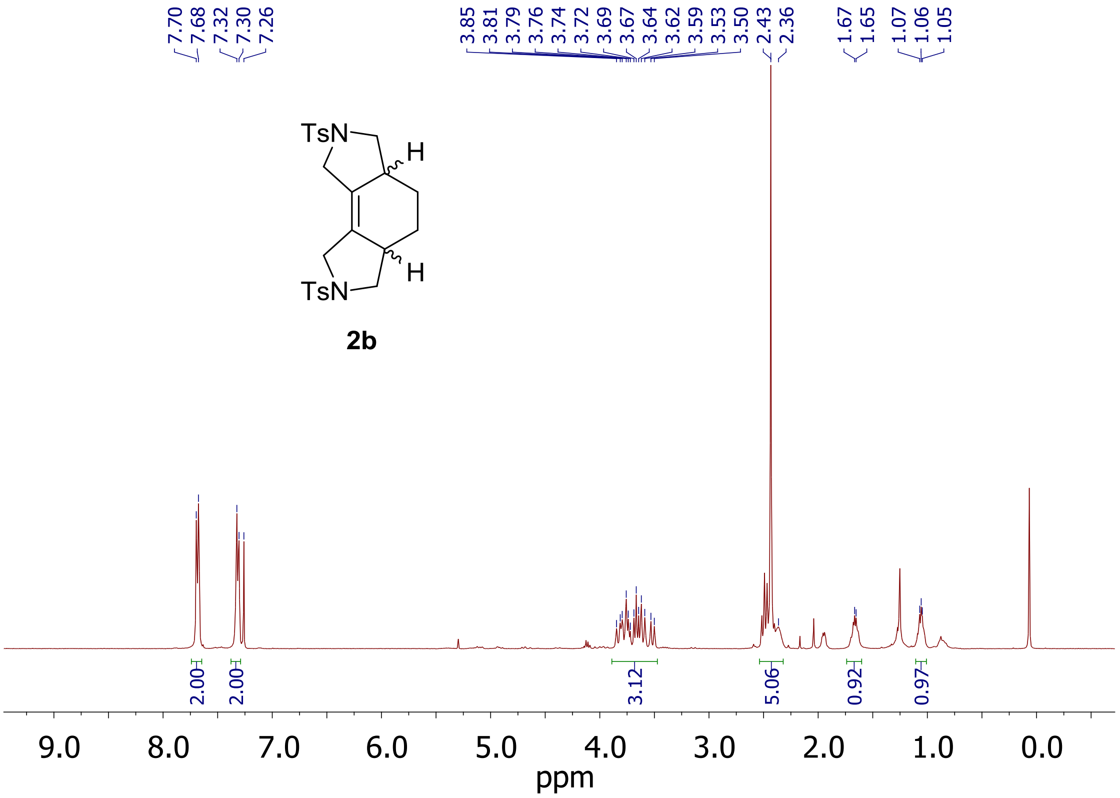

Figure S9. ^{1}H NMR spectrum of **2b** (400 MHz, $CDCl_3$).

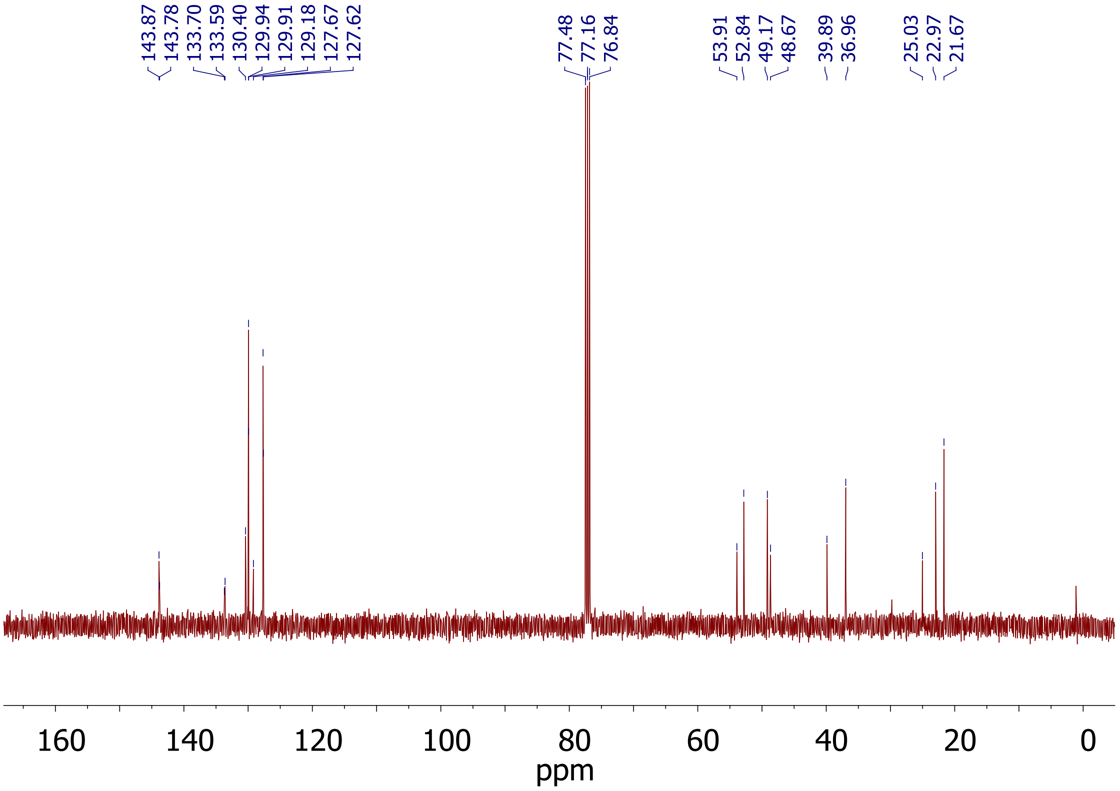

Figure S10. ^{13}C NMR spectrum of **2b** (101 MHz, $CDCl_3$).

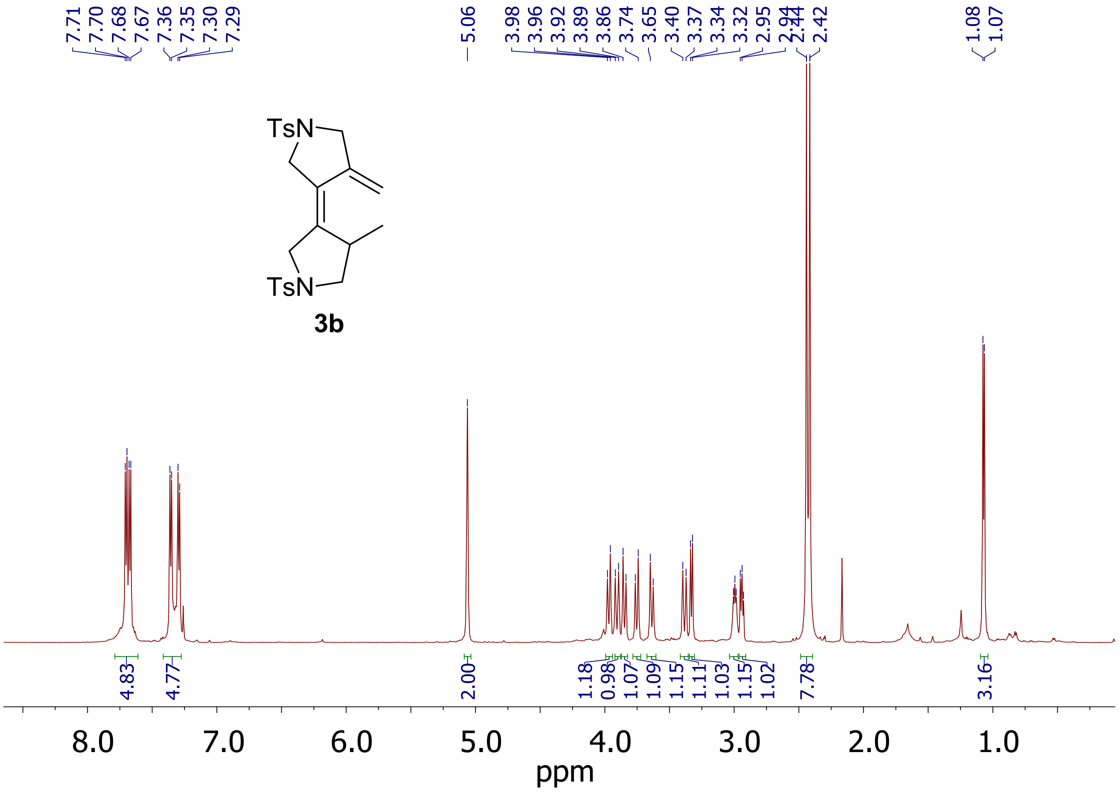

Figure S11. ^{1}H NMR spectrum of **3b** (600 MHz, $CDCl_3$).

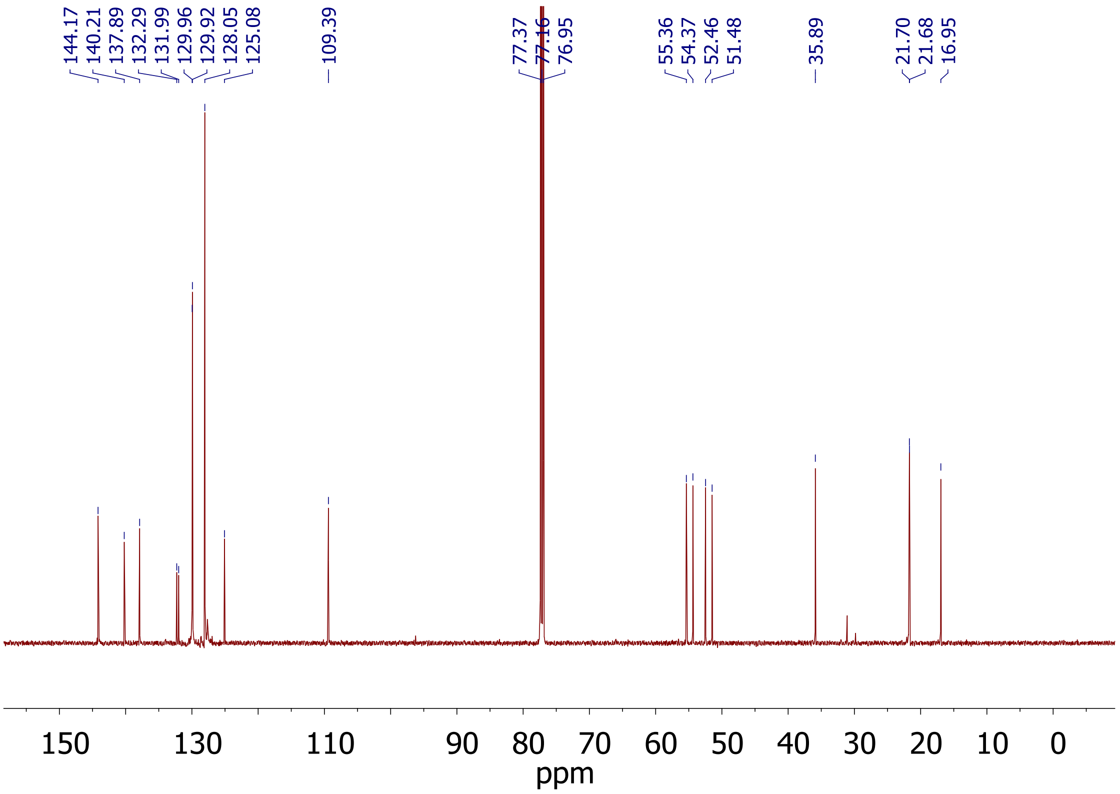

Figure S12. ^{13}C NMR spectrum of **3b** (151 MHz, $CDCl_3$).

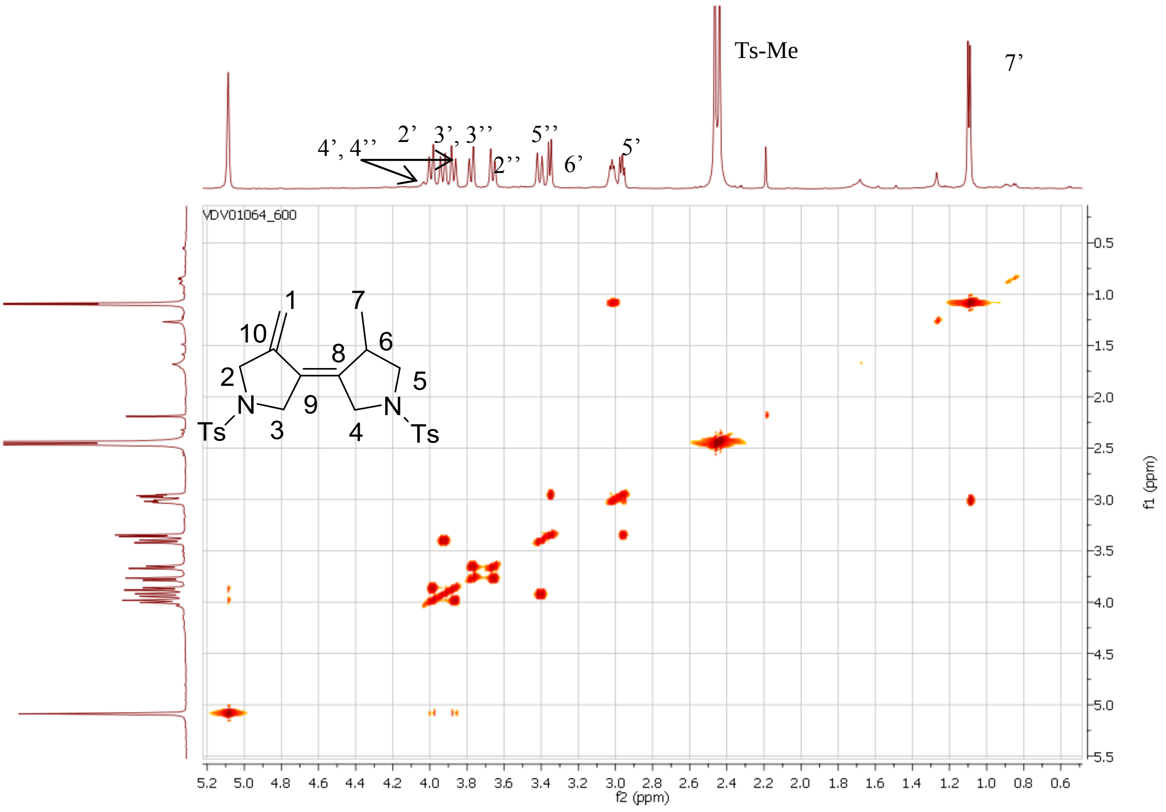

Figure S13. ^{1}H–^{1}H COSY spectrum of **3b** (600 MHz, $CDCl_3$).

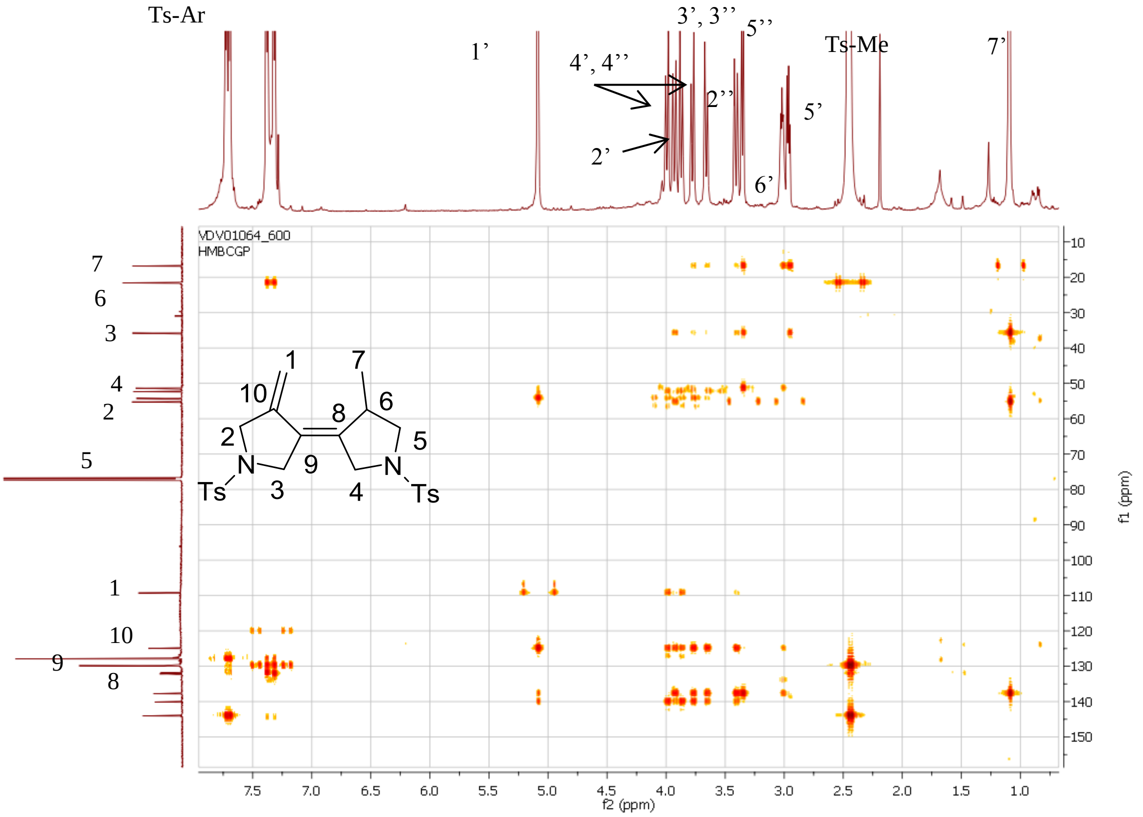

Figure S14. ^{1}H–^{13}C HMBC spectrum of **3b** (600 MHz, $CDCl_3$).

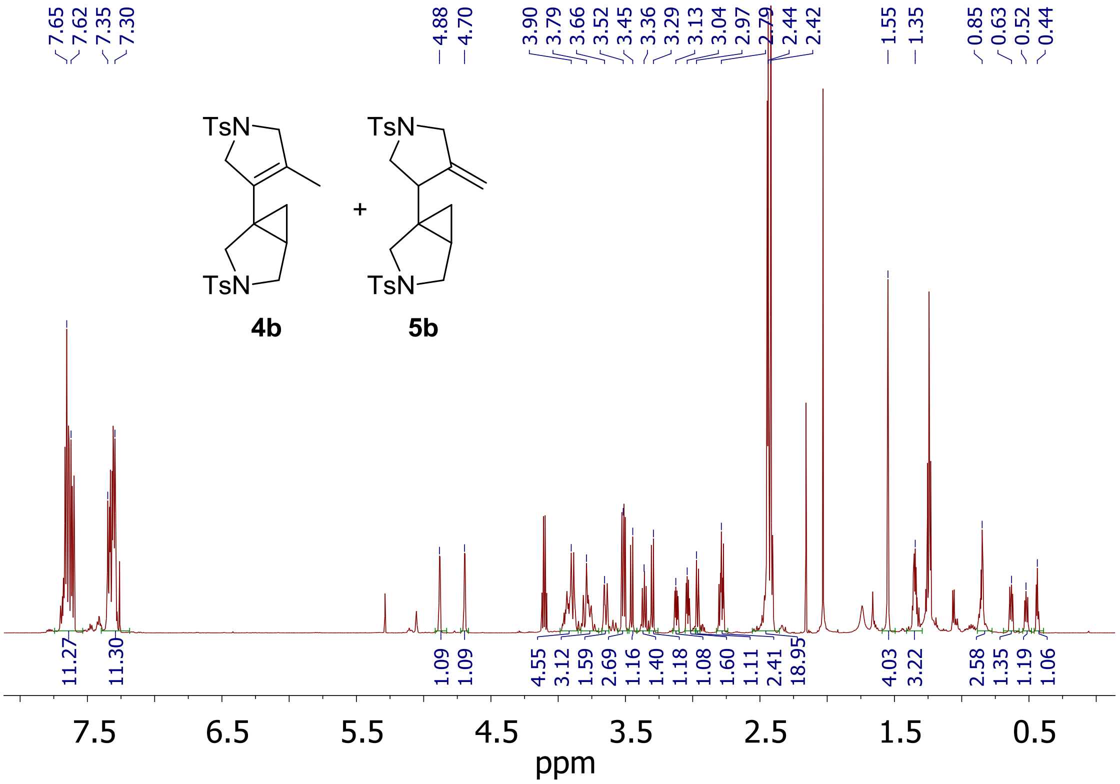

Figure S15. ^{1}H NMR spectrum of mixture **4b** and **5b** (600 MHz, $CDCl_3$).

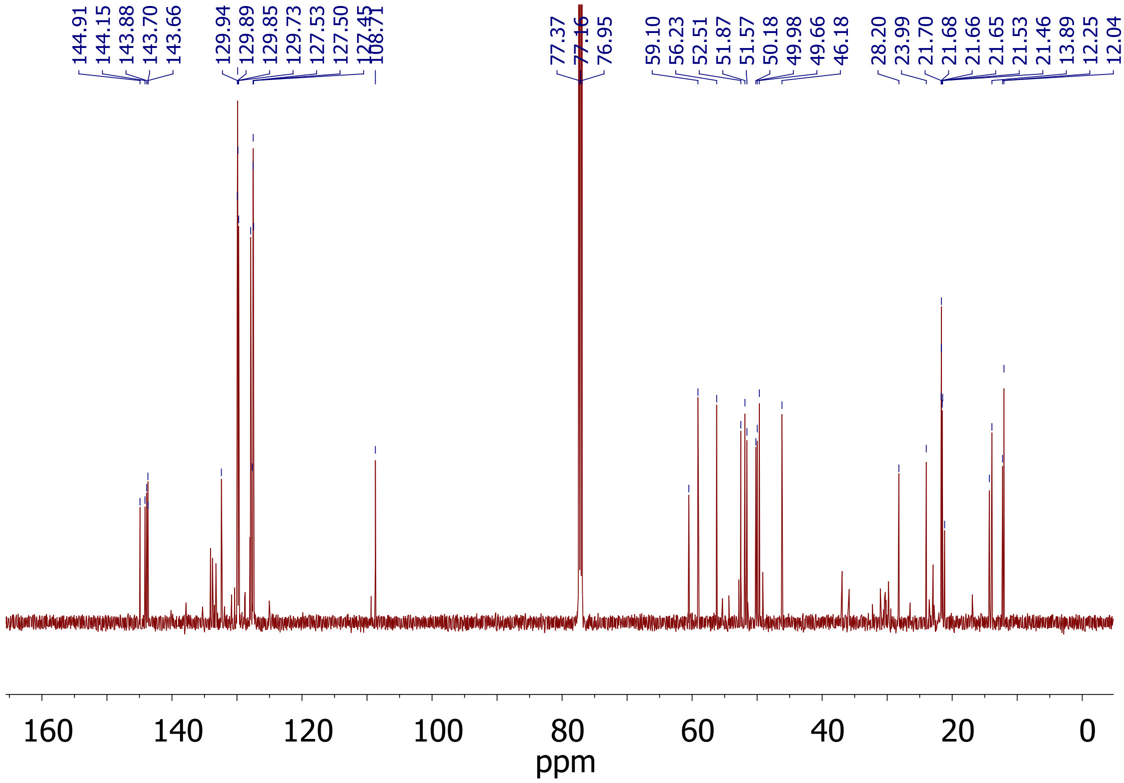

Figure S16. ^{13}C NMR spectrum of mixture **4b** and **5b** (101 MHz, $CDCl_3$).

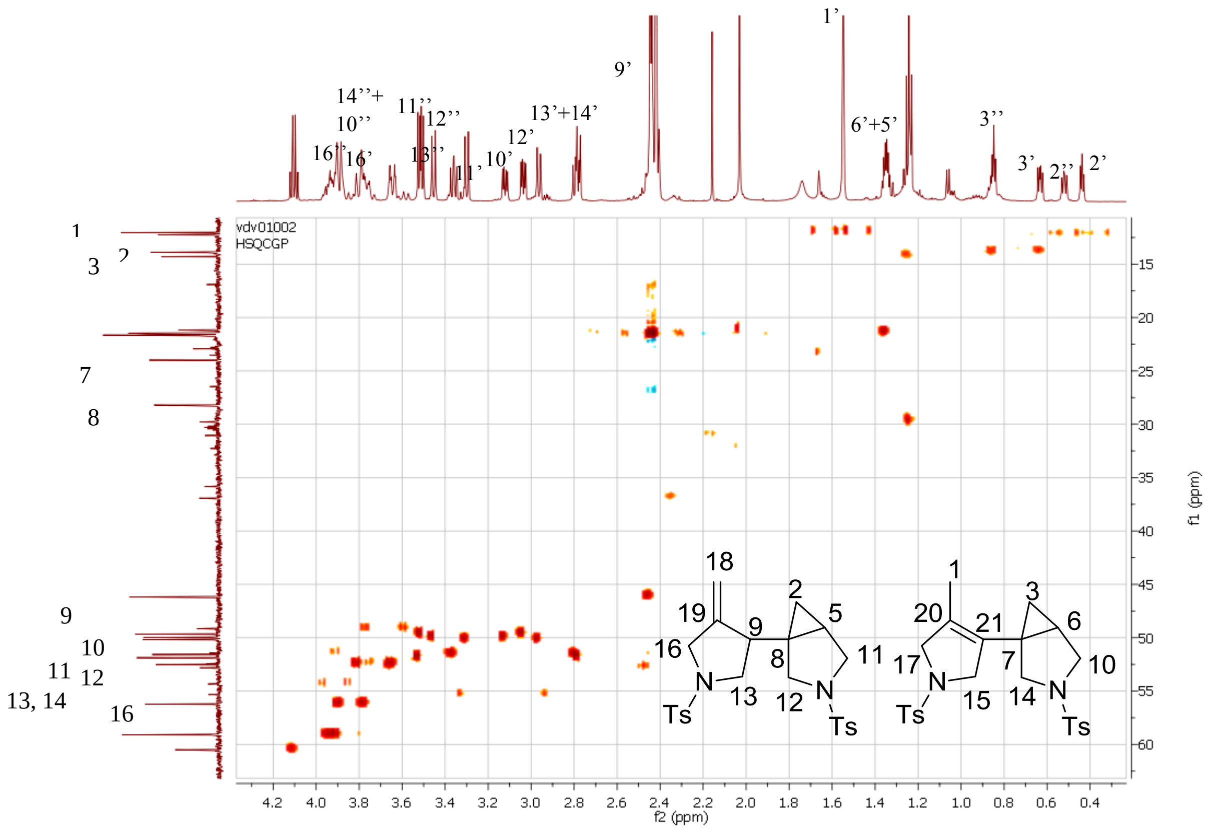

Figure S17. ^{1}H–^{13}C HSQC spectrum of mixture **4b** and **5b** (600 MHz, $CDCl_3$).

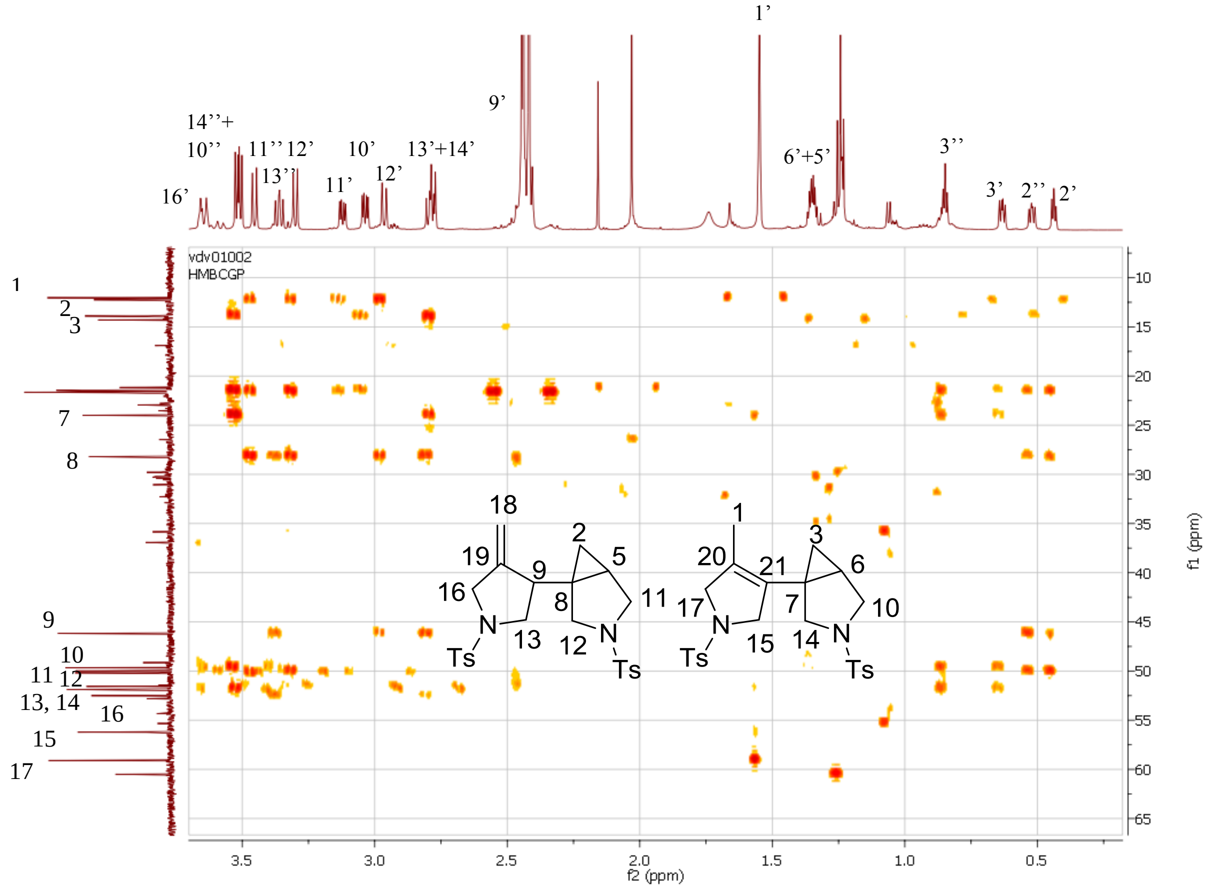

Figure S18. ^{1}H–^{13}C HMBC spectrum of mixture **4b** and **5b** (600 MHz, $CDCl_3$).

Table S1. Combined correlation NMR data for product **4b**.

	^{1}H	2'	2''	5'	9'	11'	11''	12'	12''	13'	13''	16'	16''	18'	18''
^{13}C		0.46	0.54	1.37	2.46	3.14	3.48	2.99	3.32	2.81	3.38	3.68	3.79	4.72	4.90
2	12.18	HSQC	HSQC			HMBC		HMBC	HMBC						
5	21.45	HMBC	HMBC	HSQC	HMBC				HMBC						
8	28.17	HMBC	HMBC		HMBC			HMBC	HMBC	HMBC	HMBC				
9	46.10				HSQC										
11	49.91			HMBC		HSQC	HSQC							HMBC	HMBC
12	50.13				HMBC			HSQC	HSQC						
13	51.51				HMBC					HSQC	HSQC	HMBC		HMBC	HMBC
16	52.43									HMBC	HMBC	HSQC	HSQC	HMBC	HMBC
18	108.64				HMBC							HMBC	HMBC	HSQC	HSQC
19	144.89											HMBC	HMBC	HMBC	HMBC

- HSQC, - HMBC.

Table S2. Combined correlation NMR data for product **5b**.

	^{1}H	1'	3'	3''	6'	10'	10''	14'	14''	15'	15''	17'	17''
^{13}C		1.57	0.65	0.87	1.37	3.06	3.54	2.80	3.53	3.90	3.92	3.92	3.94
1	11.96	HSQC											
3	13.81		HSQC	HSQC	HMBC	HMBC	HMBC						
6	21.10		HMBC	HMBC	HSQC	HMBC	HMBC						
7	23.95	HMBC	HMBC	HMBC			HMBC	HMBC					
10	49.59		HMBC	HMBC	HMBC	HSQC	HSQC						
14	51.79		HMBC	HMBC				HSQC	HSQC	HMBC	HMBC		
15	56.16	HMBC		HMBC						HSQC	HSQC		
17	59.02	HMBC										HSQC	HSQC
20	127.65	HMBC	HMBC	HMBC				HMBC		HMBC	HMBC	HMBC	HMBC
21	132.64	HMBC								HMBC	HMBC	HMBC	HMBC

- HSQC, - HMBC.

X-ray experimental details and structures

Crystals of **1h** ($C_{28}H_{36}N_2O_4S_2$, M = 528.71) are triclinic, space group P-1, at 120 K: a = 9.1158(6), b = 9.4944(7), c = 9.9154(7) Å, α = 61.5480(10), β = 71.9710(10), γ = 72.6980(10)°, V = 705.83(9) $Å^3$, Z = 1 (Z’ = 0.5), d_{calc} = 1.244 gcm^{-3}, μ(MoKα) = 2.24 cm^{-1}, F(000) = 282. Crystals of **2b** ($C_{24}H_{28}N_2O_4S_2$, M = 472.60) are triclinic, space group P-1, at 120 K: a = 7.5018(12), b = 12.743(2), c = 13.473(2) Å, α = 65.335(4), β = 79.309(4), γ = 86.703(4)°, V = 1149.9(3) $Å^3$, Z = 2 (Z’ = 1), d_{calc} = 1.365 gcm^{-3}, μ(MoKα) = 2.66 cm^{-1}, F(000) = 500. Intensities of 8566 and 18071 reflections for **1h** and **2b**, respectively, were measured with a Bruker APEX2 CCD diffractometer using graphite monochromated Mo-Kα radiation (*λ* = 0.71073 Å, ω-scans); 3755 and 4520 independent reflections [R_{int} 0.0244 and 0.1036] were used in further refinement for **1h** and **2b**, respectively. The structures were solved by the direct method and refined by the full-matrix least-squares against F^2 in anisotropic approximation for non-hydrogen atoms. The positions of hydrogen atoms were calculated, and they were refined in the isotropic approximation using the riding model. For **1h**, the refinement converged to wR2 = 0.1102 and GOF = 1.003 for all the independent reflections (R1 = 0.0439 was calculated against F for 3283 observed reflections with I>2σ(I)). For **2b,** the refinement converged to wR2 = 0.2060 and GOF = 1.078 for all the independent reflections (R1 = 0.0822 was calculated against F for 2614 observed reflections with I>2σ(I))**.** All calculations were performed using the SHELXTL PLUS 5.0 software (G.M. Sheldrick, Acta Cryst. A 64 (2008), 112). CCDC 1516680 and 1516681 contain the supplementary crystallographic data, which can be obtained free of charge from The Cambridge Crystallographic Data Centre *via* www.ccdc.cam.ac.uk/data_request/cif.

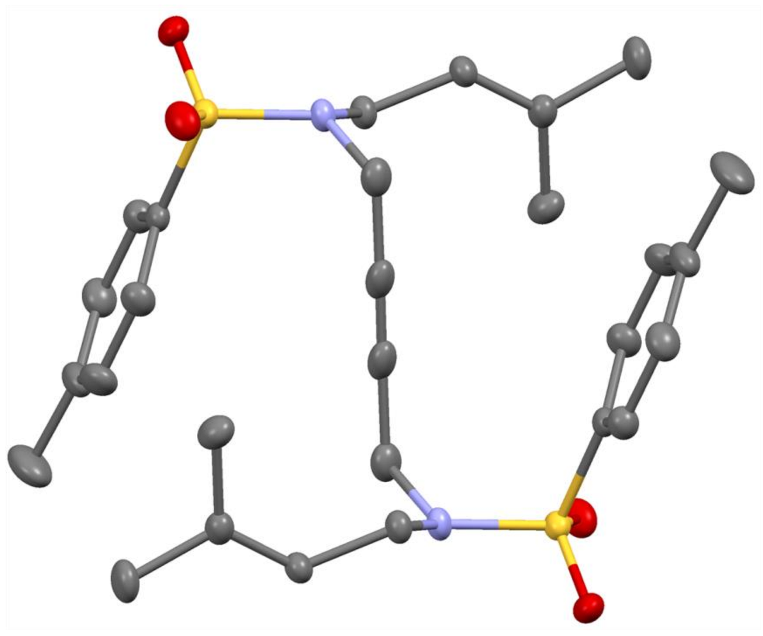

Figure 1S. The structure of the dienyne **1h** in 50% thermal elipsoids. All hydrogen atoms are omitted for clarity.

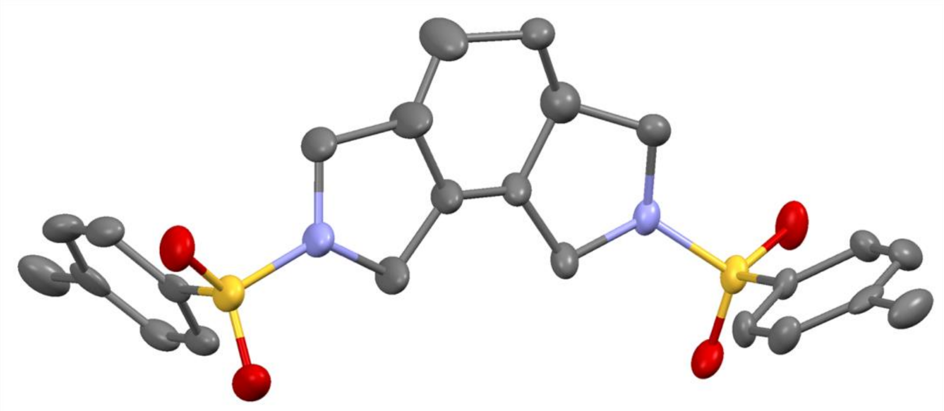

Figure 2S. The structure of the cyclohexene **2b** in 50% thermal elipsoids. All hydrogen atoms are omitted for clarity.

Calculated structure of the suggested key intermediate 6

Several isomers of **6** were calculated (including the one without double bond coordination) in order to find the most stable one. Geometry optimizations were performed using PBE exchange-correlation functional and all-electron triple-ζ basis set 3z (similar to TZ2P; H {5s1p}/[3s1p], C {11s6p2d}/[6s3p2d], O {11s6p2d}/[6s3p2d], P {15s,11p,2d}/[10s,6p,2d], Co {17s,13p,8d}/[12s,9p,4d], Br {18s,14p,9d}/[13s,10p,5d]) as implemented in the Priroda 6 code (D.N. Laikov, Yu.A. Ustynyuk, R*uss. Chem. Bull.* **2005**, 54, 820). Frequency calculations were performed to confirm the nature of the stationary point. The molecular visualization was done by ChemCraft software (http://www.chemcraftprog.com).

Table 1S. Cartesian coordinates for the optimized structure of (dppe)CoBr($C_{10}H_{14}O_2$) (**6**).

Co	-11.212214970	-8.974482740	2.768041420
Br	-10.562749120	-10.922060430	4.383083700
P	-13.388240650	-9.416198360	3.551837460
P	-11.578866700	-10.695141640	1.337647610
C	-9.479029840	-8.624380610	1.726872280
C	-9.644066150	-7.879110290	0.618561300
C	-10.997256490	-7.321411530	0.298245150
C	-11.892747900	-7.515732510	1.515220220
C	-8.083192380	-8.900985840	2.237288980
C	-8.635803640	-7.160857210	-0.247433050
C	-10.584210680	-5.894784780	-0.107940260
H	-11.429641190	-7.796085400	-0.595523490
H	-12.923764220	-7.730167660	1.218404120
H	-11.299208180	-5.380529570	-0.764678020
H	-11.900304240	-6.610769060	2.144323260
H	-10.405374090	-5.270963780	0.791578820
O	-9.366291750	-6.068269690	-0.857601190
O	-8.011974940	-9.131129730	3.635279160
C	-8.689876510	-8.132375070	4.400749800
H	-8.902371040	-8.605140020	5.367461710
H	-8.003590600	-7.274696990	4.565804870
C	-9.967596710	-7.559102800	3.804781210
H	-9.827082610	-6.708434100	3.138904620
C	-11.200050490	-7.656759530	4.448167050
H	-11.296625630	-8.260499330	5.348569800
H	-11.945298890	-6.872195630	4.311915740
H	-8.220371470	-7.773618590	-1.064905770
H	-7.786294600	-6.769527560	0.347387340
H	-7.431876070	-8.040220410	1.959117980
H	-7.640403250	-9.794635250	1.771934200
C	-13.167584170	-11.610990830	1.824989430
H	-13.906583570	-11.394784930	1.043487540
H	-12.952479180	-12.686086460	1.780716010
C	-13.686237400	-11.220678980	3.205511340
H	-13.114027890	-11.735512990	3.986815510
H	-14.748367740	-11.470546410	3.339538220
C	-10.382609040	-12.101700740	1.088441460
C	-9.124003390	-12.161412320	1.695777370
C	-10.779064320	-13.147218420	0.235174110
C	-8.271792000	-13.242146900	1.445581190
C	-9.930639780	-14.229489620	-0.003912370
C	-8.670318420	-14.278087180	0.599341190
H	-8.828705280	-11.392639570	2.403617730
H	-11.748980300	-13.114998940	-0.262639190
H	-7.296473070	-13.275598370	1.932476710
H	-10.255247770	-15.032428890	-0.666854480
H	-8.005837060	-15.122678660	0.413052450
C	-11.920231530	-10.281999080	-0.446464360
C	-10.881966870	-10.347538030	-1.390908440
C	-13.175576470	-9.803963980	-0.856240350
C	-11.098432040	-9.950887180	-2.711933930
C	-13.392118820	-9.416387770	-2.181155890
C	-12.354064120	-9.486854870	-3.113355570
H	-9.897333600	-10.702787810	-1.090954630
H	-13.992509460	-9.697149280	-0.141975080
H	-10.279198950	-10.002167250	-3.429812520
H	-14.372817880	-9.043879830	-2.479494970
H	-12.521207670	-9.178253540	-4.145641990
C	-13.971702050	-9.266639520	5.324486810
C	-15.192357220	-8.648693300	5.647443800
C	-13.204102840	-9.823877970	6.363292410
C	-15.628698390	-8.585040750	6.974184750
C	-13.649056390	-9.760417930	7.685476050
C	-14.860109690	-9.138829290	7.999009340
H	-15.814853460	-8.215127370	4.868122350
H	-12.259539900	-10.316707090	6.125790120
H	-16.579240400	-8.100175070	7.200852460
H	-13.039376800	-10.203766800	8.474014650
H	-15.203769740	-9.090007870	9.032936960
C	-14.783880390	-8.547014360	2.677740440
C	-15.787480730	-9.234333960	1.978617740
C	-14.849359180	-7.142673930	2.727529850
C	-16.820135690	-8.539464790	1.338920770
C	-15.884797140	-6.449781640	2.100171140
C	-16.873191880	-7.146131660	1.397769030
H	-15.782176080	-10.322482320	1.930609570
H	-14.084272500	-6.582376980	3.264749730
H	-17.588549790	-9.095063020	0.799892750
H	-15.917830910	-5.361388760	2.158135550
H	-17.680084560	-6.605162580	0.902846300

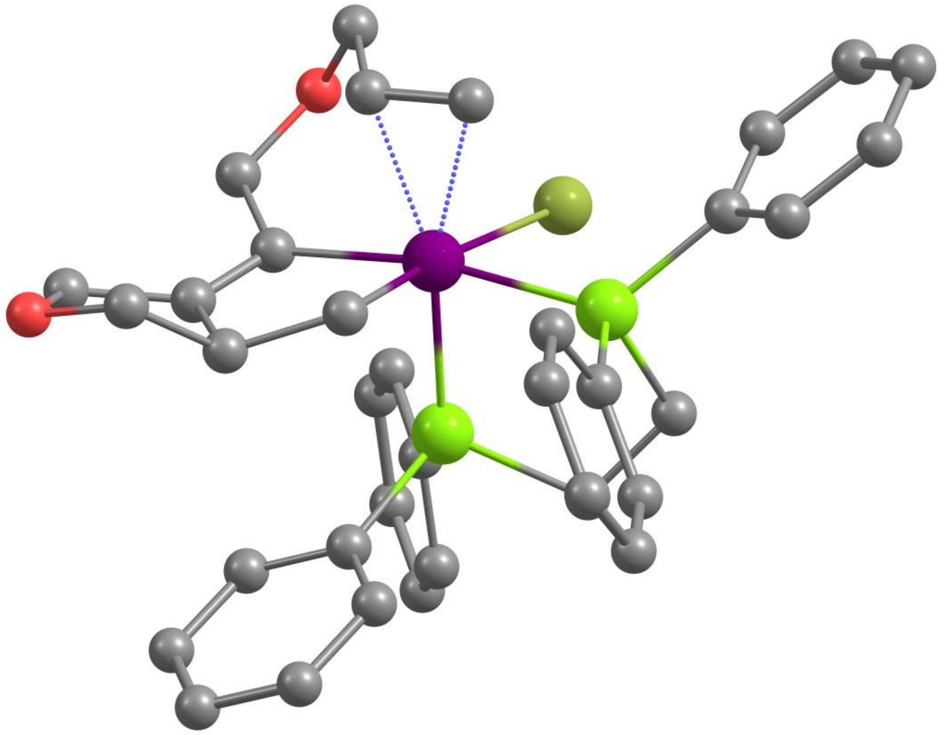

Figure 3S. The optimized structure of (dppe)CoBr($C_{10}H_{14}O_2$) (**6**). All hydrogen atoms are omitted for clarity. Violet – Co, olive – Br, green – P, red – O, grey – C.

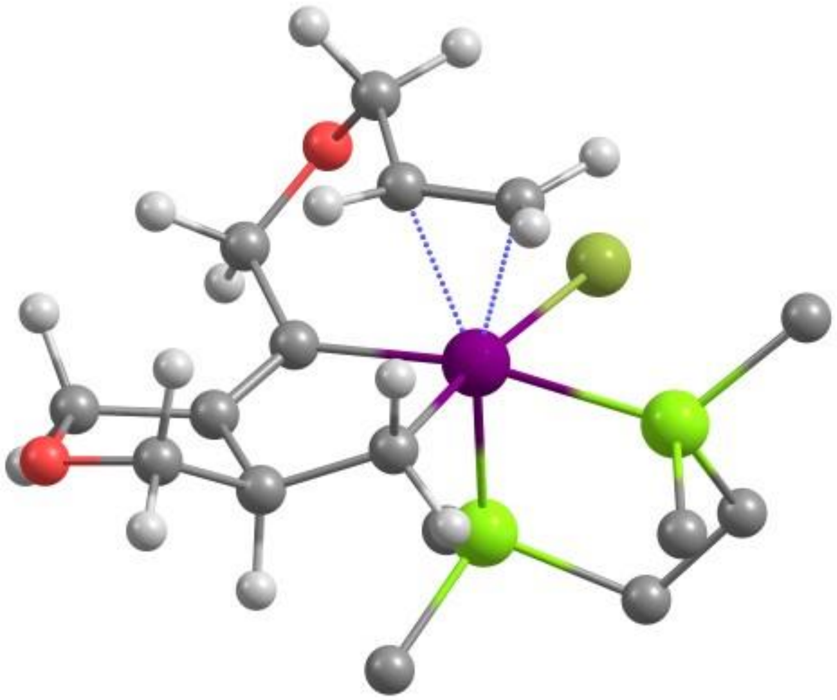

Figure 4S. The optimized structure of (dppe)CoBr($C_{10}H_{14}O_2$) (**6**). The phenyl groups and the hydrogen atoms of dppe ligand are omitted for clarity. Violet – Co, olive – Br, green – P, red – O, grey – C, white - H.
