# Supplementary material for: Unpredictable cycloisomerization of 1,11-dien-6-ynes by a common cobalt catalyst
Source: Beilstein J Org Chem. 2017 Mar 31;13:639–43. doi: 10.3762/bjoc.13.62 (PMC5389203; doi:10.3762/bjoc.13.62)

checkCIF/PLATON report

Structure factors have been supplied for datablock(s) 1d

THIS REPORT IS FOR GUIDANCE ONLY. IF USED AS PART OF A REVIEW PROCEDURE FOR PUBLICATION, IT SHOULD NOT REPLACE THE EXPERTISE OF AN EXPERIENCED CRYSTALLOGRAPHIC REFEREE.

No syntax errors found. CIF dictionary Interpreting this report

Datablock: 1d

```
Bond precision:  C-C = 0.0028 A          Wavelength=0.71073

Cell:           a=9.1158(6)       b=9.4944(7)      c=9.9154(7)
                alpha=61.548(1)   beta=71.971(1)   gamma=72.698(1)
Temperature:    120 K

                Calculated                Reported
Volume          705.83(9)                 705.83(9)
Space group     P -1                      P -1
Hall group      -P 1                      -P 1
Moiety formula  C28 H36 N2 O4 S2          C28 H36 N2 O4 S2
Sum formula     C28 H36 N2 O4 S2          C28 H36 N2 O4 S2
Mr              528.71                    528.71
Dx,g cm-3       1.244                     1.244
Z               1                         1
Mu (mm-1)       0.224                     0.224
F000            282.0                     282.0
F000'           282.36
h,k,lmax        12,12,13                  12,12,13
Nref            3758                      3755
Tmin,Tmax       0.960,0.967               0.388,0.433
Tmin'           0.925

Correction method= # Reported T Limits: Tmin=0.388 Tmax=0.433
AbsCorr = MULTI-SCAN

Data completeness= 0.999          Theta(max)= 28.996

R(reflections)= 0.0439( 3283)     wR2(reflections)= 0.1102( 3755)

S = 1.003                 Npar= 166
```

The following ALERTS were generated. Each ALERT has the format
test-name_ALERT_alert-type_alert-level.
Click on the hyperlinks for more details of the test.

Alert level C

```
PLAT906_ALERT_3_C Large K value in the Analysis of Variance ......      4.231 Check
```

Alert level G

```
PLAT154_ALERT_1_G The s.u.'s on the Cell Angles are Equal ..(Note)      0.001 Degree
PLAT380_ALERT_4_G Incorrectly? Oriented X(sp2)-Methyl Moiety .....        C14 Check
PLAT912_ALERT_4_G Missing # of FCF Reflections Above STh/L=  0.600          3 Note
PLAT960_ALERT_3_G Number of Intensities with I  <   - 2*sig(I) ...          2 Check
PLAT978_ALERT_2_G Number C-C Bonds with Positive Residual Density           8 Note
```

```
   0 ALERT level A = Most likely a serious problem - resolve or explain
   0 ALERT level B = A potentially serious problem, consider carefully
   1 ALERT level C = Check. Ensure it is not caused by an omission or oversight
   5 ALERT level G = General information/check it is not something unexpected

   1 ALERT type 1 CIF construction/syntax error, inconsistent or missing data
   1 ALERT type 2 Indicator that the structure model may be wrong or deficient
   2 ALERT type 3 Indicator that the structure quality may be low
   2 ALERT type 4 Improvement, methodology, query or suggestion
   0 ALERT type 5 Informative message, check
```

It is advisable to attempt to resolve as many as possible of the alerts in all categories. Often the minor alerts point to easily fixed oversights, errors and omissions in your CIF or refinement strategy, so attention to these fine details can be worthwhile. In order to resolve some of the more serious problems it may be necessary to carry out additional measurements or structure refinements. However, the purpose of your study may justify the reported deviations and the more serious of these should normally be commented upon in the discussion or experimental section of a paper or in the "special_details" fields of the CIF. checkCIF was carefully designed to identify outliers and unusual parameters, but every test has its limitations and alerts that are not important in a particular case may appear. Conversely, the absence of alerts does not guarantee there are no aspects of the results needing attention. It is up to the individual to critically assess their own results and, if necessary, seek expert advice.

Publication of your CIF in IUCr journals

A basic structural check has been run on your CIF. These basic checks will be run on all CIFs submitted for publication in IUCr journals (*Acta Crystallographica*, *Journal of Applied Crystallography*, *Journal of Synchrotron Radiation*); however, if you intend to submit to *Acta Crystallographica Section C* or *E* or *IUCrData*, you should make sure that full publication checks are run on the final version of your CIF prior to submission.

Publication of your CIF in other journals

Please refer to the *Notes for Authors* of the relevant journal for any special instructions relating to CIF submission.

PLATON version of 11/08/2016; check.def file version of 04/08/2016

Datablock 1d - ellipsoid plot

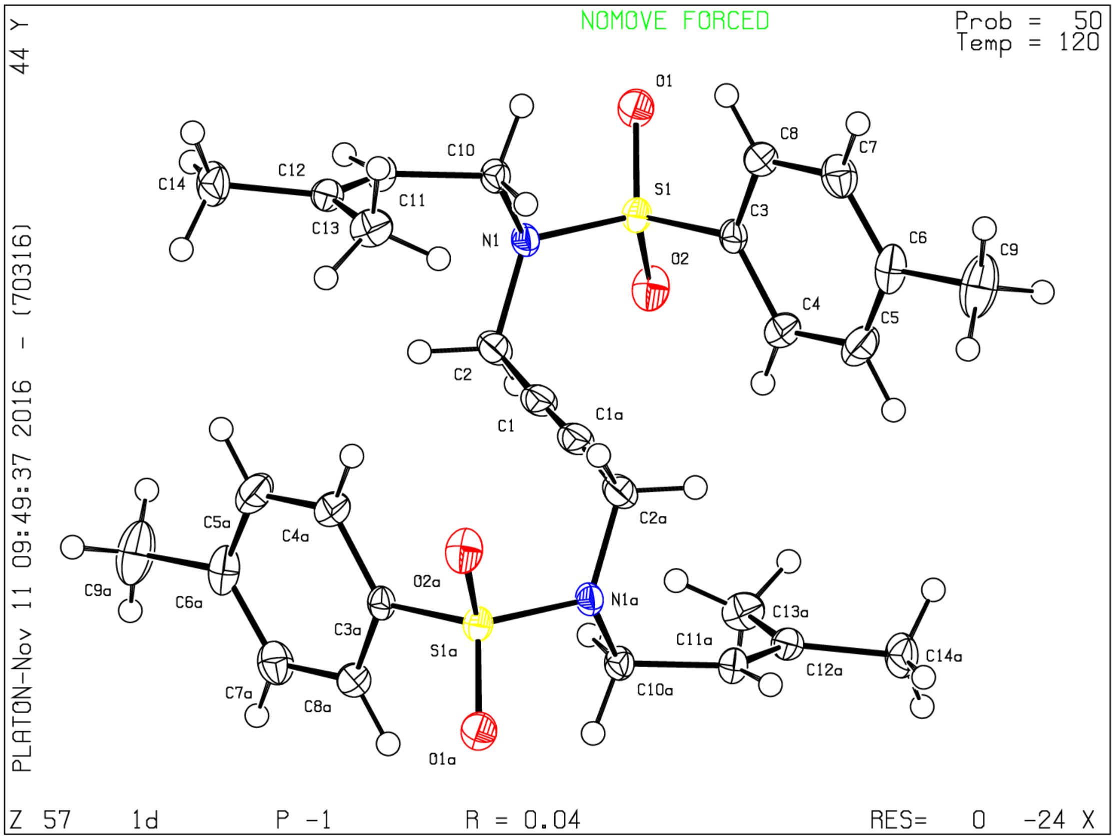

Supplement: File 2 — Single crystal data for 1h and 2b. [file Beilstein_J_Org_Chem-13-639-s002.zip › checkcif_1h.pdf]
