# Supplementary material for: Unpredictable cycloisomerization of 1,11-dien-6-ynes by a common cobalt catalyst
Source: Beilstein J Org Chem. 2017 Mar 31;13:639–43. doi: 10.3762/bjoc.13.62 (PMC5389203; doi:10.3762/bjoc.13.62)

checkCIF/PLATON report

Structure factors have been supplied for datablock(s) 2b

THIS REPORT IS FOR GUIDANCE ONLY. IF USED AS PART OF A REVIEW PROCEDURE FOR PUBLICATION, IT SHOULD NOT REPLACE THE EXPERTISE OF AN EXPERIENCED CRYSTALLOGRAPHIC REFEREE.

No syntax errors found. CIF dictionary Interpreting this report

Datablock: 2b

```
Bond precision:  C-C = 0.0073 A          Wavelength=0.71073

Cell:          a=7.5018(12)      b=12.743(2)      c=13.473(2)
               alpha=65.335(4)   beta=79.309(4)   gamma=86.703(4)
Temperature:   120 K

                Calculated                Reported
Volume          1149.9(3)                 1149.9(3)
Space group     P -1                      P -1
Hall group      -P 1                      -P 1
Moiety formula  C24 H28 N2 O4 S2          C24 H28 N2 O4 S2
Sum formula     C24 H28 N2 O4 S2          C24 H28 N2 O4 S2
Mr              472.60                    472.60
Dx,g cm-3       1.365                     1.365
Z               2                         2
Mu (mm-1)       0.266                     0.266
F000            500.0                     500.0
F000'           500.70
h,k,lmax        9,15,16                   9,15,16
Nref            4520                      4520
Tmin,Tmax       0.923,0.961               0.599,0.746
Tmin'           0.923

Correction method= # Reported T Limits: Tmin=0.599 Tmax=0.746
AbsCorr = MULTI-SCAN

Data completeness= 1.000          Theta(max)= 25.995

R(reflections)= 0.0822( 2614)     wR2(reflections)= 0.2060( 4520)

S = 1.078                  Npar= 291
```

The following ALERTS were generated. Each ALERT has the format
test-name_ALERT_alert-type_alert-level.
Click on the hyperlinks for more details of the test.

Alert level C

```
PLAT094_ALERT_2_C Ratio of Maximum / Minimum Residual Density ....       2.46 Report
PLAT220_ALERT_2_C Non-Solvent Resd 1   C   Ueq(max)/Ueq(min) Range        3.4 Ratio
PLAT241_ALERT_2_C High   'MainMol' Ueq as Compared to Neighbors of         C4 Check
PLAT241_ALERT_2_C High   'MainMol' Ueq as Compared to Neighbors of         C5 Check
PLAT241_ALERT_2_C High   'MainMol' Ueq as Compared to Neighbors of         C7 Check
PLAT242_ALERT_2_C Low    'MainMol' Ueq as Compared to Neighbors of         C6 Check
PLAT250_ALERT_2_C Large U3/U1 Ratio for Average U(i,j) Tensor ....        2.2 Note
PLAT340_ALERT_3_C Low Bond Precision on  C-C Bonds ...............    0.00733 Ang.
PLAT360_ALERT_2_C Short  C(sp3)-C(sp3) Bond   C4     -   C5     ..       1.41 Ang.
PLAT906_ALERT_3_C Large K value in the Analysis of Variance ......      4.525 Check
PLAT978_ALERT_2_C Number C-C Bonds with Positive Residual Density           0 Note
```

Alert level G

```
PLAT154_ALERT_1_G The s.u.'s on the Cell Angles are Equal ..(Note)      0.004 Degree
PLAT343_ALERT_2_G Unusual sp3      Angle Range in Main Residue for     C4     Check
PLAT793_ALERT_4_G The Model has Chirality at C4      (Centro SPGR)          R Verify
PLAT793_ALERT_4_G The Model has Chirality at C7      (Centro SPGR)          R Verify
PLAT910_ALERT_3_G Missing # of FCF Reflection(s) Below Theta(Min)           1 Note
```

0 **ALERT level A** = Most likely a serious problem - resolve or explain
0 **ALERT level B** = A potentially serious problem, consider carefully
11 **ALERT level C** = Check. Ensure it is not caused by an omission or oversight
5 **ALERT level G** = General information/check it is not something unexpected

1 ALERT type 1 CIF construction/syntax error, inconsistent or missing data
10 ALERT type 2 Indicator that the structure model may be wrong or deficient
3 ALERT type 3 Indicator that the structure quality may be low
2 ALERT type 4 Improvement, methodology, query or suggestion
0 ALERT type 5 Informative message, check

It is advisable to attempt to resolve as many as possible of the alerts in all categories. Often the minor alerts point to easily fixed oversights, errors and omissions in your CIF or refinement strategy, so attention to these fine details can be worthwhile. In order to resolve some of the more serious problems it may be necessary to carry out additional measurements or structure refinements. However, the purpose of your study may justify the reported deviations and the more serious of these should normally be commented upon in the discussion or experimental section of a paper or in the "special_details" fields of the CIF. checkCIF was carefully designed to identify outliers and unusual parameters, but every test has its limitations and alerts that are not important in a particular case may appear. Conversely, the absence of alerts does not guarantee there are no aspects of the results needing attention. It is up to the individual to critically assess their own results and, if necessary, seek expert advice.

Publication of your CIF in IUCr journals

A basic structural check has been run on your CIF. These basic checks will be run on all CIFs submitted for publication in IUCr journals (*Acta Crystallographica*, *Journal of Applied Crystallography*, *Journal of Synchrotron Radiation*); however, if you intend to submit to *Acta Crystallographica Section C* or *E* or *IUCrData*, you should make sure that full publication checks are run on the final version of your CIF prior to submission.

Publication of your CIF in other journals

Please refer to the *Notes for Authors* of the relevant journal for any special instructions relating to CIF submission.

PLATON version of 11/08/2016; check.def file version of 04/08/2016

Datablock 2b - ellipsoid plot

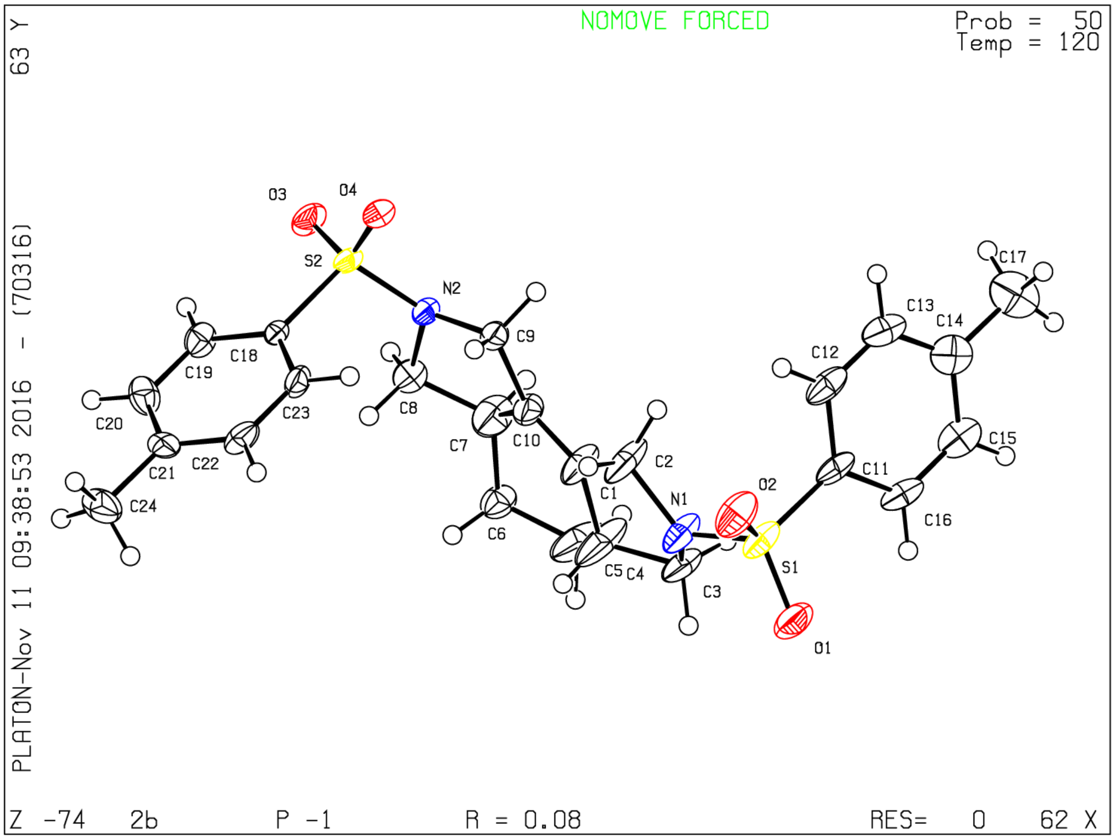

Supplement: File 2 — Single crystal data for 1h and 2b. [file Beilstein_J_Org_Chem-13-639-s002.zip › checkcif_2b.pdf]
